# Imaging the neurovascular unit in health and neurodegeneration: a scoping review of interdependencies between MRI measures

**DOI:** 10.1186/s12987-023-00499-0

**Published:** 2023-12-21

**Authors:** Ella Rowsthorn, William Pham, Mohammad-Reza Nazem-Zadeh, Meng Law, Matthew P. Pase, Ian H. Harding

**Affiliations:** 1 Department of Neuroscience, Central Clinical School, Monash University, 99 Commercial Road, Melbourne, VIC 3004 Australia; 2 Turner Institute for Brain and Mental Health & School of Psychological Sciences, Monash University, 18 Innovation Walk, Clayton, VIC 3168 Australia; 3 Department of Radiology, Alfred Health, 99 Commercial Road, Melbourne, VIC 3004 Australia; 4 Department of Electrical and Computer Systems Engineering, Monash University, 14 Alliance Lane, Clayton, VIC 3168 Australia; 5 Harvard T.H. Chan School of Public Health, 677 Huntington Avenue, Boston, MA 02115 USA; 6 Monash Biomedical Imaging, Monash University, 762-772 Blackburn Road, Clayton, VIC 3168 Australia

**Keywords:** Neurovascular unit, Blood–brain barrier, Glymphatic system, Perivascular space, Cerebral blood flow, Free water, White matter hyperintensities, Neurodegenerative disease, Magnetic resonance imaging

## Abstract

The neurovascular unit (NVU) is a complex structure that facilitates nutrient delivery and metabolic waste clearance, forms the blood–brain barrier (BBB), and supports fluid homeostasis in the brain. The integrity of NVU subcomponents can be measured in vivo using magnetic resonance imaging (MRI), including quantification of enlarged perivascular spaces (ePVS), BBB permeability, cerebral perfusion and extracellular free water. The breakdown of NVU subparts is individually associated with aging, pathology, and cognition. However, how these subcomponents interact as a system, and how interdependencies are impacted by pathology remains unclear. This systematic scoping review identified 26 studies that investigated the inter-relationships between multiple subcomponents of the NVU in nonclinical and neurodegenerative populations using MRI. A further 112 studies investigated associations between the NVU and white matter hyperintensities (WMH). We identify two putative clusters of NVU interdependencies: a ‘vascular’ cluster comprising BBB permeability, perfusion and basal ganglia ePVS; and a ‘fluid’ cluster comprising ePVS, free water and WMH. Emerging evidence suggests that subcomponent coupling within these clusters may be differentially related to aging, neurovascular injury or neurodegenerative pathology.

**Supplementary Information:**

The online version contains supplementary material available at 10.1186/s12987-023-00499-0.

## Background

### The neurovascular unit in health and disease

The neurovascular unit (NVU) is a complex structure which regulates the bidirectional transport of fluid, metabolic products and other molecules between the periphery and the brain, ensuring efficient neural function and maintaining a healthy brain environment. The cellular components of the NVU interact and dynamically respond to local metabolic demands, neuroimmune processes and activity of neurons that synapse with astrocytes of the NVU to regulate blood flow, fluid exchange, glucose and oxygen delivery, and metabolic waste removal [1].

**Fig. 1:**
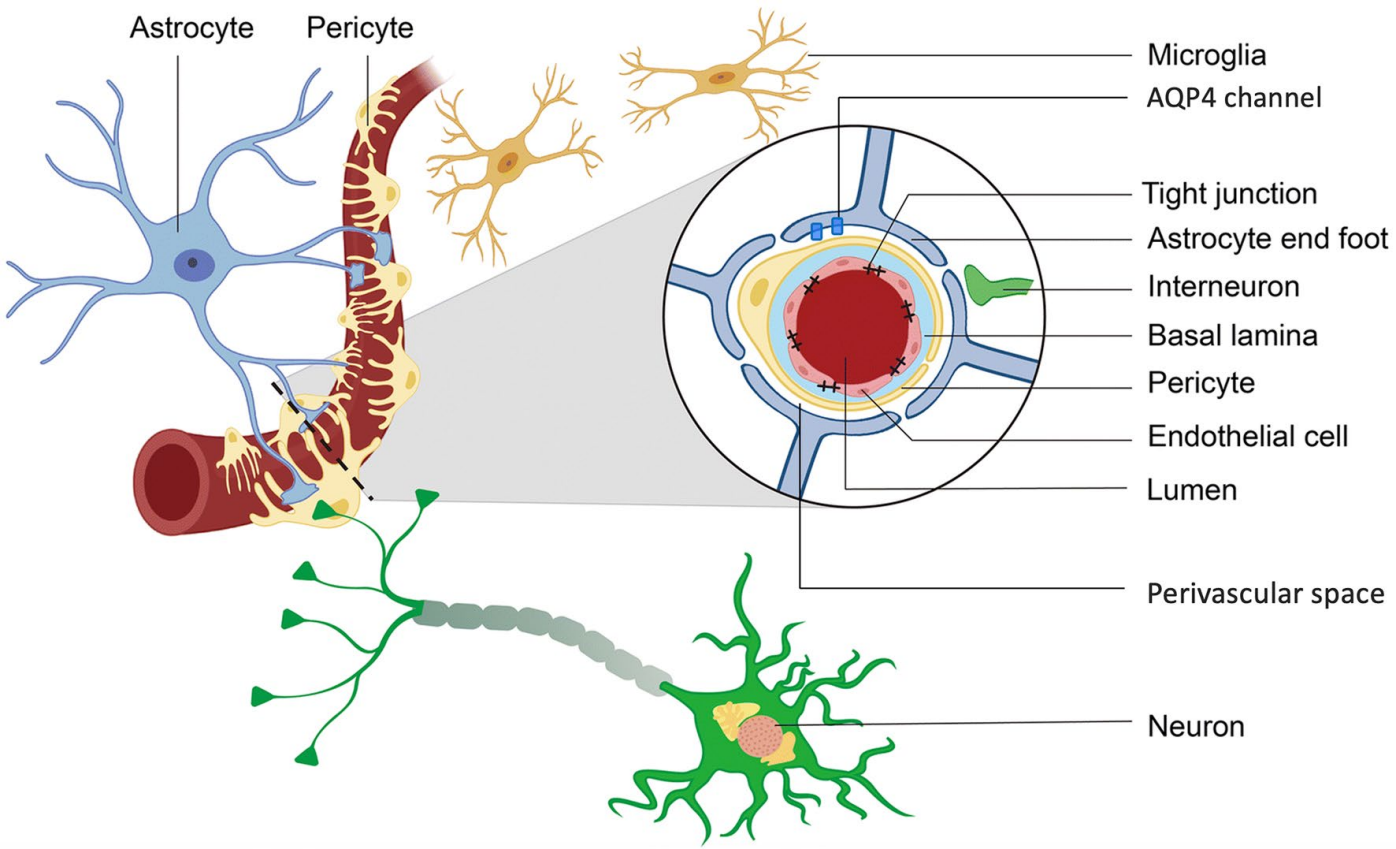
The Neurovascular Unit. The neurovascular unit is the multifaceted structure in the brain that facilitates blood supply, metabolic waste clearance and protection from potentially harmful peripheral material. From inwards to outwards, it is comprised of the artery which supplies blood, the endothelial cell and tight junctions that form the blood–brain barrier, the perivascular space which supports glymphatic waste clearance, and the astrocytes that synapse onto parenchymal neurons and host aquaporin-4 (AQP4) channels that facilitate fluid exchange. Adapted with permission from Sun et al. [157]

At the level of penetrating arteriole (i.e., small vessels), the NVU is comprised of several key components or sub-structures [2]. At the centre of the NVU, the blood vessel is crucial for the continuous supply of oxygen and nutrients to the brain. Surrounding the vessel, endothelial cells and tight junctions form the blood–brain barrier (BBB) responsible for protecting the brain from potentially harmful peripheral material. Smooth muscle cells (or pericytes towards the level of the capillary) surround the endothelial cells and regulate vessel dilation/constriction to ensure local metabolic demands are met with temporal precision. A cerebrospinal fluid (CSF)-filled perivascular space surrounds the endothelial basement membrane (basal lamina) and is thought to facilitate the CSF flow and clearance of metabolic waste from the brain. Lastly, the perivascular space is encompassed by astrocyte end-feet which facilitate fluid exchange through the NVU including via aquaporin-4 (AQP4) channels (Fig. 1).

A great deal of literature has broadly identified NVU impairment as a feature of many neurodegenerative diseases [3, 4], with these changes identified in the earliest stages of many neurodegenerative cascades [5, 6]. However, how these subcomponents interact as a system, and how interdependencies are impacted by pathology remains unclear. Recent advancements in magnetic resonance imaging (MRI) methodology have provided valuable tools for investigating the structural and functional changes of different components in the NVU in the context of both healthy aging and neurodegenerative disease.

### The vessel and cerebral blood flow

The vessel’s continuous delivery of blood is essential for meeting the metabolic demands of neural activity and supporting all brain structures. Blood flow in the brain is moderated by multiple adaptive processes in the NVU including endothelial cell- and pericyte-facilitated vasodilation and vasoconstriction [7, 8], microglial activity [9] and astrocyte-mediated neurovascular coupling [10]. Hypoperfusion, or decreased cerebral blood flow (CBF), has been associated with accelerated cognitive decline, increased risk of dementia conversion and greater Alzheimer’s disease biomarker severity [11–13].

CBF and cerebral blood volume can be measured using arterial spin labelling (ASL) MRI. Briefly, ASL is the process by which a bolus of blood is magnetically ‘tagged’ within the carotid artery just before entering the brain [14, 15]. The distribution of this tagged blood across the brain is then measured several seconds later, allowing quantification of the local perfusion rate and volume of blood perfused [14, 15]. Cerebrovascular reactivity (CVR), defined as the change in CBF in response to a contextual manipulation (e.g., administered CO_2_ or prescribed behavioural change), is a dynamic marker of neurovascular coupling [16]. Poorer CVR is observed in ischemic stroke, cardiovascular disease and Alzheimer’s disease, and is associated with risk of cognitive impairment progression [17, 18].

### The BBB and permeability

Encompassing the vessel is the BBB, the interface between the circulatory system and central nervous system that is fundamental for facilitating nutrient supply to the brain and protecting it from harmful pathogens. The BBB is selectively semi-permeable to molecules that pass through tight junctions in the endothelial membrane of the NVU. BBB permeability can be modulated by endothelial proteins and by inflammatory responses involving pericytes, microglia and astrocytes [19–22]. Consequently, a loss of essential tight junction proteins and expression impairs selective permeability, which is thought to increase transport of potentially neurotoxic molecules through the BBB, contributing to neurodegenerative pathology [22–24].

BBB permeability is most commonly assessed using dynamic contrast-enhanced MRI (DCE MRI) as a marker of BBB structural integrity. DCE MRI measures the change in magnetic resonance signal between the intravascular and extravascular compartments of the brain due to an injected agent, such as a gadolinium-based contrast. Due to its molecular size, gadolinium cannot pass through tight junctions of the BBB unless their functioning is impaired. As such, BBB integrity can be imaged by quantifying the transfer, or ‘leakage’, of contrast from the vasculature through to the extracellular space. DCE MRI is typically quantified using a pharmacokinetic equation to quantify the transfer coefficient which is sensitive to low-level permeability and therefore can detect subtle changes in BBB permeability, commonly denoted as K^trans^ or K_i_ [25, 26]. An increase in BBB permeability, or a higher transfer coefficient, is associated with cerebral small vessel (CSVD), mild cognitive impairment and Alzheimer’s disease [27–29].

### The perivascular space and MR-visible enlargement

Surrounding the structures comprising the BBB, perivascular spaces are the fluid-filled passageways that encapsulate vasculature within the NVU and are the primary component of the brain’s glymphatic system [30]. Perivascular spaces facilitate CSF and interstitial fluid exchange important for metabolic waste clearance and prevention of neurotoxic protein accumulation [31, 32]. Although their specific causes are undetermined, enlargement of the perivascular space is often considered an indicator of glymphatic dysfunction or stagnation in the NVU [31, 32].

When enlarged due to an increase in CSF volume, perivascular spaces are visible on T1- or T2-weighted MRI due their high water content and can be quantified as a count (the number of perivascular spaces identified) or volume (the total sum of voxels identified, in mm^3^). MRI-visible enlarged perivascular spaces (ePVS) are most prevalent in the basal ganglia (BG) and the centrum semiovale (CSO) but can be observed throughout the white matter of the brain. A higher number or greater volume of ePVS is associated with stroke, neuroinflammation and neurodegenerative pathologies such as Alzheimer’s disease [30, 33].

### Astrocyte AQP4 channels and extracellular free water

AQP4 channels are expressed on astrocyte endfeet which enclose the perivascular space. These channels facilitate passive transport of water molecules through the NVU, supporting the exchange of fluid between the perivascular space and interstitial fluid within the surrounding brain parenchyma [34, 35]. Free water refers to water molecules that are directionally unrestricted (isotropic diffusion) within the white matter of the brain. Extracellular free water is thought to possibly increase as a component of neuroinflammatory response [36–38], stagnation of glymphatic drainage [39–41], and/or reduced density of the parenchymal cellular matrix (i.e., loss of neurons or neurites) and is thus likely a composite marker of neurodegeneration, neuroinflammation and vascular leakage. While the presence of free water is a ubiquitous part of normal brain homeostasis and increases are observed in normal aging, abnormal accumulation of extracellular free water may be a sensitive, early marker of pathology, particularly with respect to white matter microstructures [42, 43]. Notably, increased free water has been associated with cerebral small vessel disease (CSVD) [44], Parkinson’s disease [45] and Alzheimer’s disease [46], and is thought to precede formation of white matter hyperintensities (WMH) [47]. Normal-appearing white matter conversion to WMH is predicted by increased baseline free water levels [47], and furthermore, the relationship between cognitive decline and baseline WMH has been shown to be mediated by baseline free water [48].

Local accumulation of extracellular fluid, or free water, in the brain parenchyma can be measured using diffusion-weighted MRI as isotropic diffusing water molecules. Conventional diffusion tensor imaging approaches quantify mean diffusivity (or apparent diffusion coefficient) which is reflective of total fluid diffusion in a region. More specific measures of the free water fraction can also now be derived using biophysical modelling of the diffusion signal. These approaches include bi-tensor free water models (such as Pasternak) and three-compartment models (such as neurite orientation dispersion and density imaging, NODDI). Markedly elevated free water levels have been associated with Parkinson’s disease progression, multiple sclerosis degeneration and biomarkers of Alzheimer’s disease [49–51]. Finally, WMH are seen in T2 fluid-attenuated inversion recovery (FLAIR) MRI and are considered to be representative of white matter gliosis and degeneration [52]. As WMH also represent an increase in fluid content they may reflect a more progressed accumulation of free water.

### The missing link: understanding the NVU as an integrated system

The NVU is a complex and dynamic system that is formed by many integrated subcomponents. However, as elements of the NVU are typically measured in isolation in vivo, the dependencies between the subparts of this system in aging and neurodegenerative disease remain unclear. For example, does reduced BBB integrity co-occur with fluid transport abnormalities? Does reduced perfusion or vascular compliance impact parenchymal extracellular fluid levels or contribute to the development of white matter lesions? Questions such as these have important implications for developing mechanistic models of NVU function and dysfunction, particularly in aging or progressive neurodegenerative diseases where vascular dysfunction may cascade through interdependent components of the NVU or may accrue from pathological processes. As changes in the NVU are thought to precede, and even contribute to neuronal loss in aging and neurodegenerative disease [5], a more in-depth understanding of this system may elucidate additional mechanisms contributing to brain aging, facilitate future predication and early detection of neurodegenerative risk, and reveal opportunities to measure and track the earliest stages of neurodegenerative pathology.

The present review therefore aims to synthesize the existing literature investigating the relationships between MRI measures of NVU dysfunction. Specifically, this review will synthesize studies that investigate how different measures of NVU dysfunction are inter-related and how factors such as age, disease pathology, or brain region influence the presence or strength of these inter-relationships. Given the limited body of literature addressing diverse inter-relationships across neurodegenerative diseases, this endeavour lends itself to a systematic scoping review.

## Methods

### Search design

Four categories of search terms were defined corresponding to four respective elements of interest of the NVU: (i) ePVS, (ii) BBB permeability, (iii) brain perfusion and hemodynamics, and (iv) extracellular free water and WMH. As extracellular free water is a relatively new measure in this context and likely underrepresented in the literature, WMH were included to supplement category (iv). All categories included common synonyms and phrasing for each MRI measure to ensure all relevant literature was captured (e.g., both ‘Virchow-Robin’ and ‘perivascular space’ terms were included in the ePVS category). To capture studies which potentially investigated inter-relationships between these elements, the search was structured such that only studies that had terms from at least two different categories within the abstract, title or keywords would qualify the search (e.g., a paper which had “perivascular space” and “perfusion” would qualify, while a paper which only had “perfusion” and “blood volume” would not).

Medline, Embase and Web of Science databases were systematically searched using the defined term categories and structure. No publication date restrictions were applied. The search excluded literature tagged as a ‘review’ or exclusively tagged as an animal study. Database specific search strategies are included in the Additional file 1.

### Selection criteria

To meet screening inclusion criteria, literature needed to investigate a relationship (e.g., correlation, mediation or moderation) between at least two NVU MRI measures in humans and be available in English. As this field is rapidly progressing and some of the literature addressing the research questions were expected to be recent, gray literature (i.e., conference abstracts, conference posters and pre-prints) were included from January 2021 or later. This time frame is considered sufficient to capture research that is in the peer-review process.

Literature that studied samples of non-neurodegenerative disease patients, psychiatric patients, or those with injury/insult without a clear vascular component were excluded (i.e., epileptic disorders, cancer, developmental disorders, schizophrenia, bipolar disorder). Additionally, as the aim of this review was to investigate MRI measures representative of NVU dysfunction rather than changes resulting from specific insult, literature that exclusively investigated MRI measures in the context of pre/post-surgery change or recently acquired injury or vascular event (i.e., MRI within 6-months of insult) were also excluded.

**Fig. 2:**
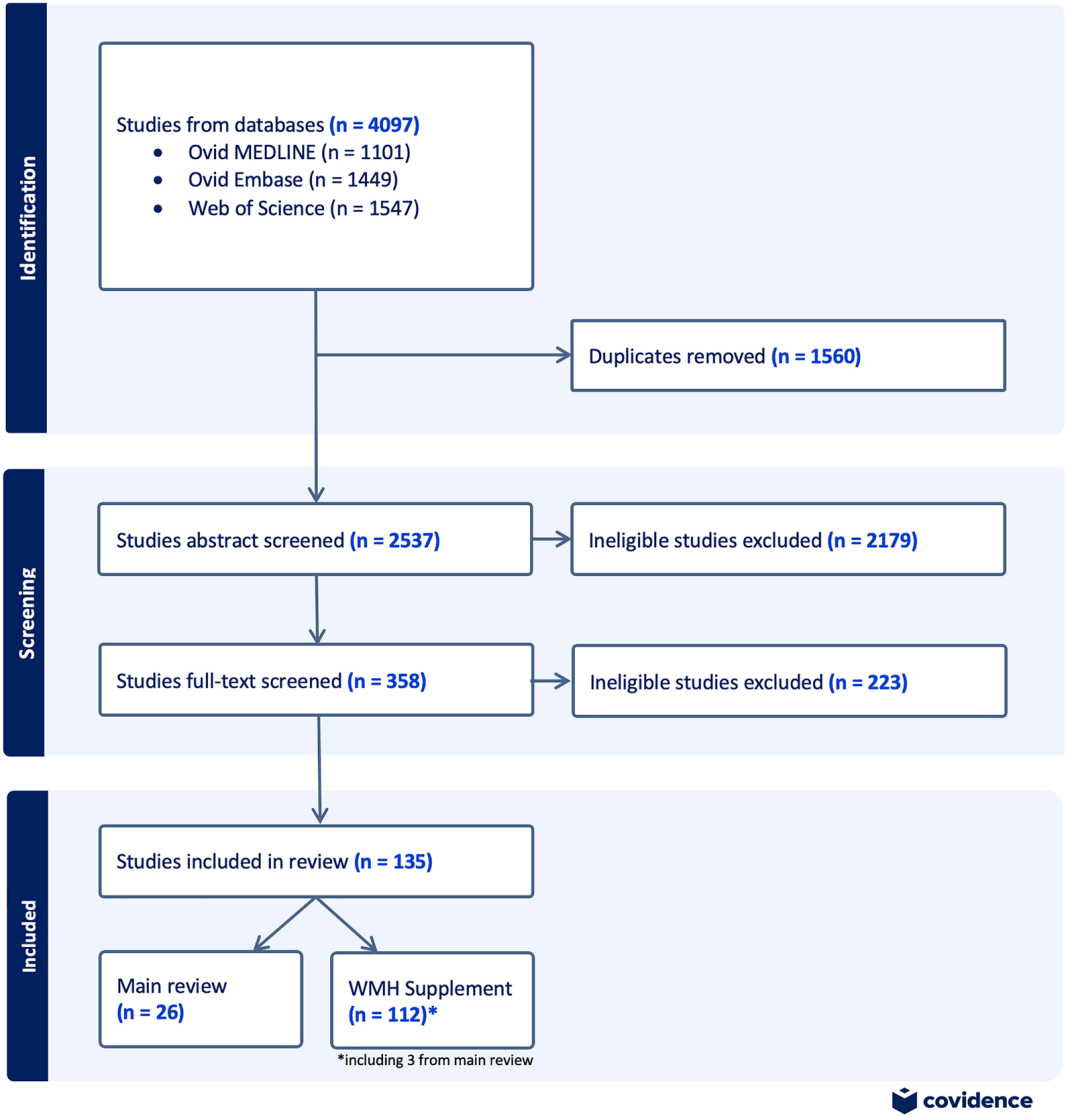
PRISMA flow diagram of literature search and screening

Search results from all three databases were imported in to Covidence for review. Abstracts were first independently double-screened by ER (reviewer 1) and WP or MRNZ (reviewer 2). Any discrepancies between reviewers were resolved by consensus. Full text screening was then undertaken by ER in consultation with IHH and MP. Duplicates identified during the screening process were removed (Fig. 2).

### Data extraction

Data was extracted by ER from the remaining studies, which included: information about the sample (patient type and age), MRI sequence and quantification method, inter-relationship statistical strength and significance, and a qualitative summary of findings. Extracted information was reviewed and corroborated by WP.

For this review, study samples comprised of participants that were considered representative of the general population, community-based samples or samples that do not have a specific disease or are without significant neurological disease, cognitive impairment or insult are referred to as ‘healthy’.

The amount of literature resulting from the WMH search component was substantial despite the term being included as a conceptual supplement to the extracellular free water search category. To maintain the focus of this review on the early or subtle changes of measures in NVU dysfunction, rather than resulting atrophy or late-stage fluid accumulation, literature was subsequently partitioned into main inter-relationships (e.g., ePVS vs Perfusion), and WMH relationships (eg., ePVS vs WMH). Literature which researched WMH relationships are detailed in the Additional file 1.

This review was developed and conducted in accordance to Preferred Reporting Items for Systematic reviews and Meta-Analyses extension for Scoping Reviews (PRISMA-ScR) guidelines [53]. The protocol for this review was registered online to OSF registries on February 13, 2023, and is accessible from https://doi.org/10.17605/OSF.IO/PDM54 (registration no: PDM54). The final literature search was conducted on February 6, 2023.

## Results

### Literature search

One hundred and thirty-five studies were eligible and included in this review, including 2 gray literature publications. 26 studies were included as the main review data for this paper (summarised in Table 1) and the 112 studies that addressed WMH relationships were summarised in the supplement (Additional file 1: Table S1). There were no studies which investigated more than one inter-relationship, but three of the main studies additionally investigated measure associations with WMH. Our search and screening process did not identify any relevant studies that investigated the relationship between perfusion and free water.

**Table 1:** Summary of main review study samples, MRI methodologies and relationship investigated

Author (Year)	Sample/Group (*N*)^a^	Age, M ± *SD*	MRI Method^b,c^		Inter-relationship		
ePVS × BBB permeability			ePVS	BBBP	Region	Association	Summary
Chen et al. (2022)	T2DM CSVD (*n* = 25)	69 ± 10	T1 or T2 visual count	Gd-DOTA DCE k^trans^	Global ePVS and BBBP in: frontal white matter, parietal white matter, gray matter, caudate head, putamen, thalamus	None (only with presence of cerebral microbleeds; (*statistics not reported*)	Non-significant
Chen et al. (2022)	Non-T2DM CSVD (*n* = 12)	56 ± 19	T1 or T2 visual count	Gd-DOTA DCE k^trans^	Global ePVS and BBBP in: frontal white matter	When ePVS ≥ 6, ≥ 7 or ≥ 8; *p* = 0.028 to 0.035	Positive
Chen et al. (2022)	Non-T2DM (*n* = 12)	56 ± 19	T1 or T2 visual count	Gd-DOTA DCE k^trans^	Global ePVS and BBBP in: parietal white matter, gray matter, caudate head, putamen, thalamus	None (*statistics not reported)*	Non-significant
Li et al. (2019)	Healthy (*N* = 109)	70 ± 9	T2 visual count grade	gadolinium DCE k^trans^	BG ePVS and global BBBP	When comparing low (0–1) and high (2–4) grade ePVS; *p* < 0.001	Positive
					CSO ePVS and global BBBP	When comparing low (0–1) and high (2–4) grade ePVS; *p* = 0.88	Non-significant
Li et al. (2018)	Healthy (*N* = 99) (same cohort as 2019 study)	70 ± 9	T2 visual count grade	gadolinium DCE k^trans^	Global ePVS and BBBP in: NAWM, WMH, cortical gray matter and deep gray matter	*R*^*2*^ = 0.082 to 0.214, *p* ≤ 0.001 to 0.004	Unspecified
Wardlaw et al. (2009)	Lacunar stroke (*n* = 51) & Cortical stroke (*n* = 46)	64 ± 12 69 ± 10	1.5 T T2 visual count grade	1.5 T Gd-DTPA DCE signal enhancement	Global ePVS and BBBP in: white matter, gray matter	*F* = 0.9 to 1.75, *p* = 0.13 to 0.46	Non-significant
ePVS × Cerebral perfusion			ePVS	Perfusion	Region	Association	Summary
Gyanwali et al. (2022)	All memory clinic patients (N = 333)	Median = 73 (IQR = 10)	T2 visual count	pCASL CBF	Global ePVS and CBF in: gray matter, white matter	β < 0.01, *p* = 0.45 to 0.90	Non-significant
					BG ePVS and CBF in: gray matter, white matter	β = − 0.02 to − 0.04, *p* = 0.17 to 0.52	Non-significant
					CSO ePVS and CBF in: gray matter, white matter	β < 0.01, *p* = 0.70 to 0.83	Non-significant
Gyanwali et al. (2022	Normal cognition (n = 87)	*Not reported*	T2 visual count	pCASL CBF	Global ePVS and CBF in: gray matter, white matter	β = − 0.01 to 0.00, *p* = 0.86 to 0.94	Non-significant
					BG ePVS and CBF in: gray matter, white matter	β = − 0.05 to 0.01, *p* = 0.21 to 0.83	Non-significant
					CSO ePVS and CBF in: gray matter, white matter	β = − 0.01 to 0.00, *p* = 0.59 to 0.99	Non-significant
Gyanwali et al. (2022	Cognitive impairment no dementia (n = 153)	*Not reported*	T2 visual count	pCASL CBF	Global ePVS and CBF in: gray matter, white matter	β = − 0.01, *p* = 0.30 to 0.54	Non-significant
					BG ePVS and CBF in: gray matter, white matter	β = − 0.04 to − 0.01, *p* = 0.32 to 0.73	Non-significant
					CSO ePVS and CBF in: gray matter, white matter	β = − 0.01 to 0.00, *p* = 0.79 to 0.98	Non-significant
Gyanwali et al. (2022)	Dementias (n = 93)	*Not reported*	T2 visual count	pCASL CBF	Global ePVS and CBF in: gray matter, white matter	β = − 0.01 to 0.01, *p* = 0.54 to 0.74	Non-significant
					BG ePVS and CBF in: gray matter, white matter	β = − 0.03 to 0.00, *p* = 0.58 to 0.92	Non-significant
					CSO ePVS and CBF in: gray matter, white matter	β = 0.01 to 0.03, *p* = 0.25 to 0.83	Non-significant
Kapoor et al. (2022)	Healthy (*n* = *37*)	66 ± 7	T2 visual count grade	pCASL CVR to hypercapnia/ hypocapnia	Global	Hypocapnia: *B* = − 2.02, *p* = 0.015 Hypocapnia: *B* = − 0.33, *p* = 0.871	Non-significant Non-significant
					BG ePVS and global CVR	Hypercapnia: *B* = − 2.7, *p* = 0.009 Hypocapnia: None* (statistics not reported)*	Negative Non-significant
					CSO ePVS and global CVR	Hypercapnia: *B* = − 1.8, *p* = 0.068 Hypocapnia: None* (statistics not reported)*	Non-significant Non-significant
Lee et al. (2021)	Spontaneous ICH (*n* = 21)	63 ± 11	1.5 T T2 visual count	1.5 T pulsed ASL CVR to dipyridamole	Global	*p* > 0.05	Non-significant
Liu et al. (2021)	Healthy (N = 406)	70 ± 8	T2 visual count grade (BG only)	pCASL CBF	BG ePVS and CBF in: cortical regions and left thalamus	When comparing mild (grade < 3) and severe (grade ≥ 3) ePVS: *p* < 0.05	Negative
Lu et al. (2022)	CSVD (n = 121) Healthy (n = 53)	64 ± 9 57 ± 9	T2 visual count grade	pCASL CBF pattern category (discriminative analysis)	Total ePVS and CBF pattern	When comparing between three pattern groups: *p* > 0.05	Non-significant
					BG ePVS and CBF pattern	When comparing between three pattern groups: *p* > 0.05	Non-significant
					CSO ePVS and CBF pattern	When comparing between three pattern groups: *p* > 0.05	Non-significant
Neumann et al. (2022)	CSVD (*n* = 10) and Healthy (*n* = 4)	70 (IQR = 14) 72 (IQR = 9)	T2 visual count (BG/CSO/Hippocampus)	Pulsed ASL CBF	Total ePVS and CBF in: gray matter, white matter, basal ganglia, hippocampus	*r* = − 0.80 to − 0.39 s*ignificance value not available*	Negative
					BG ePVS and CBF in: gray matter, white matter, basal ganglia, hippocampus	*r* = − 0.82 to − 0.60 s*ignificance value not available*	Negative
					CSO ePVS and CBF in: gray matter, white matter, basal ganglia, hippocampus	*r* = − 0.49 to 0.15 s*ignificance value not available*	Non-significant
Onkenhout et al. (2020)	Vascular cognitive impairment (N = 132)	73 ± 10	T2 visual count grade	phase-contrast CBF	Global	When comparing mild (grade < 3) and severe (grade ≥ 3) ePVS (*adjusted difference* = 3.3, *p* = 0.1)	Non-significant
						When ePVS severe (grade ≥ 3) (*OR* = 0.67, *p* = 0.03)	Negative
Shi et al. (2020)	Minor stroke (N = 56)	68 ± 8	1.5 T T1, T2 visual count grade	1.5 T phase-contrast CBF	BG ePVS and global CBF	*OR* = 1.01–1.03, s*ignificance not available, ORs overlap null*	Non-significant
					CSO ePVS and global CBF	*OR* = 0.99–1.00, s*ignificance not available, ORs overlap null*	Non-significant
ePVS × Free water			ePVS	Free water	Region	Association	Summary
Huang et al. (2022)	CSVD (*n* = 84)	61 ± 11	T2 visual count grade	DTI ISOVF	Global	β = 0.224, *p* < 0.001	Positive
Huang et al. (2022)	Healthy (*n* = 144)	61 ± 6	T2 3D volume	DTI ISOVF	Global	β = 0.290, *p* < 0.001	Positive
Huang et al. (2021)	Healthy (N = 136)	Median = 60 (range 56–65)	T2 3D non-WMH volume	DTI non-PVS ISOVF	Global	β = 0.332, *p* < 0.001	Positive
						High-WMH (above median): ePVS × WMH is mediated by free water (β = 0.118, *p* < 0.006)	Unspecified
						Low-WMH (below median): ePVS × WMH is not mediated by free water (β = − 0.046, *p* = 0.074)	Non-significant
Jiaerken et al. (2021)	Severe CSVD (*n* = 15)	67 (range 57–81)	T2 visual label location	DTI ISOVF	ePVS voxels and local free water	Compared to NAWM, free water is higher in (0–2 mm; *p* < 0.001) and lower 3–4 mm from ePVS (*p* < 0.001)	Unspecified
Jiaerken et al. (2021)	Healthy Elderly (*n* = 20)	64 (range 56–83)	T2 3D segmentation	DTI ISOVF	ePVS voxels and local free water	Compared to NAWM, free water is higher in (0–2 mm; *p* < 0.001) and lower 2–4 mm from ePVS (*p* < 0.001)	Unspecified
Jiaerken et al. (2021)	Healthy Adults (*n* = 28)	32 (range 20–59)	T2 3D segmentation	DTI ISOVF	ePVS voxels and local free water	Compared to NAWM, free water is higher in (0–2 mm; *p* < 0.001) and lower 3–6 mm from ePVS (*p* < 0.001)	Unspecified
Lan et al. (2022)	CSVD (N = 129)	60 ± 11	1.5 T T1, T2 visual count grade (BG only)	1.5 T DTI ISOVF	BG ePVS and global free water	*r* = 0.428, *p* < 0.001; β = 0.154, *p* = 0.045	Positive
Zotin et al. (2022; gray literature)	Probable CAA (N = 38)	73 ± 7	Not reported	DTI ISOVF	CSO ePVS and global free water	β = 0.496, *p* < 0.001	Positive
BBB permeability × Cerebral perfusion			BBBP	Perfusion	Region	Association	Summary
Chen et al. (2022; gray literature)	Asymptomatic carotid artery stenosis (*N* = *30*)	Not reported	DCE k^trans^	ASL perfusion (not reported)	Cerebral hemisphere with carotid artery stenosis	Areas with increased BBBP also had reduced perfusion, *statistics not available*	Negative
Chi et al. (2019)	SLE (*n* = *6*)	38 ± 13	gadabutrol DCE k^trans^ and volume in extravascular extracellular space (V_e_)	gadabutrol DCE CBF	Global	k^trans^: *r* = 0.47, R^2^ = 0.22, *p* = < 0.001	Positive
						V_e_: *r* = 0.47, R^2^ = 0.23, *p* = < 0.001	Positive
Chi et al. (2019)	Healthy (*n* = *5*)	34 ± 11	gadabutrol DCE k^trans^ and volume in extravascular extracellular space (V_e_)	gadabutrol DCE CBF	Global	k^trans^: *r* = 0.22, *p* > 0.05	Non-significant
						V_e_: *r* = 0.25, *p* > 0.05	Non-significant
Haselhorst et al. (2000)	Multiple sclerosis (*N* = *25*)	43 (range 22–61)	1.5 T Gd-DOTA enhancing lesion locations	1.5 T Gd-DOTA regional CBV	CBV in DCE enhancing lesions and white matter	When comparing enhancing lesions to NAWM, *p* < .01	Positive
van de Haar et al. (2016)	MCI/AD (*n* = *14*)	75 (range 65–85)	gadobutrol DCE influx (K_i_) and fraction of leaking voxels (V_L_)	pCASL CBF	Gray matter BBBP and gray matter CBF	K_i_: r = − 0.73, *p* = 0.011	Negative
						V_L_: r = − 0.57, *p* = 0.07	Non-significant
van de Haar et al. (2016)	Healthy (*n* = *16*)	77 (range 65–85)	gadobutrol DCE influx (K_i_) and fraction of leaking voxels (V_L_)	pCASL CBF	Gray matter BBBP and gray matter CBF	K_i_: *p* = 0.60	Non-significant
						V_L_: *p* = 0.60	Non-significant
Varatharaj et al. (2019)	RR-MS (*n* = *12*) Healthy (*n* = *13*)	43 ± 10 31 ± 10	Gadovist DCE influx	Gadovist DCE CBF and CBV	Global	CBF: *rho* = 0.32, *p* = 0.11	Non-significant
						CBV: B = 0.036, *p* < 0.001	Positive
Wengler et al. (2019)	Healthy (N = 15)	28 ± 9	pCASL surface area product (PS) and extraction fraction (EF)	pCASL CBF	Global	PS: *r* = 0.89, *p* < 0.05	Positive
						EF: *r* = − 0.32, *p* < 0.05	Negative
Wong et al. (2019)	CSVD (N = 27)	69 ± 12	gadobutrol DCE influx (K_i_) and fraction of leaking voxels (V_L_)	DSC CBF	Global CBF and BBBP in: white matter	K_i_: r = − 0.40, *p* = 0.045	Negative
						V_L_: r = − 0.41, *p* = 0.033	Negative
					Global CBF and BBBP in: gray matter	K_i_: r = − 0.36, *p* = 0.094	Non-significant
						V_L_: r = − 0.37, *p* = 0.077	Non-significant
Wuerfel et al. (2004)	RR-MS (N = 20)	32 ± 9	1.5 T Gd-DPTA enhancing lesion locations	1.5 T T2^a^ CBF and CBV	CBF/CBV in DCE enhancing lesions	CBF: When comparing baseline to time of initial enhancement (*p* = 0.015) and baseline to 3-weeks before BBB leakage (17.9% increase, *p* = 0.008)	Positive
						CBV: When comparing baseline to time of initial enhancement (*p* = 0.008) and baseline to 3-weeks before BBB leakage (18.0% increase, *p* = 0.008)	Positive
BBB permeability × Free water			BBBP	Free water	Region	Association	Summary
Hillmer et al. (2022)	VCID/AD/Leukoaraiosis (N = 136)	68 (IQR = 13)	Gd-DTPA DCE k^trans^	DTI ISOVF	Global	*p* < 0.001	Positive

Measure associations are summarised as either ‘positive’, ‘negative’, ‘unspecified’ (analysis does not detail relationship direction) or ‘non-significant’ (did not meet statistical significance). Strength and statistical significance of relationship reported where available. *B* = unstandardised regression coefficient; β = standardised regression coefficient; *F* = F ratio; *p* = significance value; *OR* = odds ratio; r = Pearson correlation coefficient; *R*^*2*^ = coefficient of determination; *rho* = Spearman’s rank correlation coefficient

*AD* Alzheimer’s disease, *ASL* arterial spin labelling, *BBB* blood–brain barrier, *BBBP* blood–brain barrier permeability, *BG* basal ganglia, *CAA* cerebral amyloid angiopathy, *CBF* cerebral blood flow, *CBV* cerebral blood volume, *CSO* centrum semiovale, *CSVD* cerebral small vessel disease, *CVR* cerebrovascular reactivity, *DCE* dynamic contrast enhanced, *DSC* dynamic susceptibility contrast, *DTI* diffusion tensor imaging, *ePVS* enlarged perivascular space, *Gd-DOTA* gadoterate meglumine, *Gd-DTPA* gadolinium diethyltriamine pentaacetic acid, *ICH* intracerebral haemorrhage, *IQR* inter-quartile range, *ISOVF* isotropic volume fraction, *k*^*trans*^ volume transfer constant of contrast (leakage), *MCI* mild cognitive impairment, *NAWM* normal appearing white matter, *pCASL* pseudo-continuous arterial spin labelling, *RR-MS* relapse-remitting multiple sclerosis, *SLE* systemic lupus erythematosus, *T2DM* type-2 diabetes mellitus, *VCID* vascular cognitive impairment and dementia, *WMH* white matter hyperintensity

^a^“Healthy” refers to samples that are comprised of participants that do not have a specific disease or are without significant neurological disease, cognitive impairment or insult

^b^MRI is 3T unless otherwise specified

^c^ePVS is quantified in the BG *and* CSO unless otherwise specified. “Grade”: 0 = no visual ePVS, 1 ≤ 10, 2 = 10–20, 3 = 20–40, 4 ≥ 40

### ePVS and BBB permeability

Four studies investigated the relationship between ePVS and BBB permeability, all using T2-weighted MRI and gadolinium based DCE MRI to quantify these variables respectively.

Li et al. [54] found that ePVS severity explained a significant amount of variance in BBB permeability in multiple brain areas among a healthy cohort, including within areas of both WMH and normal-appearing white matter. Further analysis in the same cohort found that BBB permeability was only significantly different between those with low and high BG ePVS burden, while no significant difference was observed between those with low and high CSO ePVS groups [55].

Chen et al. [56] investigated whether CSVD-associated markers could predict increased BBB permeability in groups of individuals with CSVD and either with or without type-2 diabetes mellitus. They found that ePVS alone was not significantly associated with BBB permeability in individuals with type-2 diabetes mellitus and CSVD. However, in individuals with CSVD without type-2 diabetes, ePVS scores of ≥ 6 were associated with increased BBB permeability.

In contrast, Wardlaw et al. [57] reported that ePVS did not explain a significant amount of variance in BBB permeability in their sample of patients with lacunar and cortical strokes in either white matter or gray matter.

Collectively, there seems to be a consensus between the two studies of healthy individuals and studies of those with CSVD that ePVS is positively associated with BBB permeability, although this association may be specific to BG ePVS. However, in samples of patients with diabetes and stroke, no significant association between ePVS and BBB permeability was found.

### ePVS and perfusion

The inter-relationship between ePVS and perfusion has been investigated in eight studies. While all studies quantified ePVS using T2-weighted MRI, there was variability in the measure used to assess perfusion and hemodynamics, including variations of ASL and phase-contrast MRI.

Liu et al. [58] investigated the relationship between pseudo-continuous ASL CBF and BG ePVS in a healthy sample. They found a negative association between gray matter CBF and BG ePVS, with significant hypoperfusion observed in those with high grade BG ePVS in multiple cortical regions.

Neumann et al. [59] investigated the association between ePVS and pulsed ASL CBF in a sample including CSVD and healthy participants. They reported a negative correlation between ePVS and CBF, with the strongest association found in white matter. Region-specific sub-analysis indicated that BG ePVS had a stronger correlation with CBF than CSO ePVS, both in white matter and gray matter.

Lu et al. [60] studied regional CBF variability across CSVD patients and derived three sub-group categories based on discriminative spatial CBF patterns. However, ePVS severity was not significantly different between the three CBF pattern groups, whether considering BG ePVS, CSO ePVS or total ePVS severity.

Onkenhout et al. [61] investigated phase-contrast CBF in a cohort of people with vascular cognitive impairment. They found that higher CBF was significantly associated with high-grade (≥ 20 counts) ePVS burden, however, there was no significant difference in CBF between patients with low-grade ePVS (< 20 counts) and high-grade ePVS.

Further in the context of cognitive impairment, Gyanwali et al. (2022) [62] examined the relationship between ePVS in the BG and CSO and pseudo-continuous ASL CBF in patients recruited from a memory clinic. Patients were categorised into groups based on cognitive status: normal cognition, mild cognitive impairment and dementias. However, no significant relationships between ePVS and CBF were found within any group.

Shi et al. [63] did not find a significant association between either BG ePVS or CSO ePVS and phase-contrast CBF or arterial pulsatility index in patients with lacunar and cortical strokes.

Kapoor et al. [64] found a negative association between ePVS and cerebrovascular reactivity to hypercapnia but no association with reactivity to hypocapnia (paced breathing) in healthy participants.

Lee et al. [65] investigated cerebrovascular reactivity in patients with spontaneous intercranial haemorrhage but did not find a significant correlation between change in CBF response to an administered vasoconstrictor and ePVS in this group.

Overall, there was inconsistent support for a relationship between ePVS and perfusion, with some studies showing a significant negative association (where greater ePVS was associated with hypoperfusion) and others reporting non-significant results. Notably, however, this inconsistency may be explained by sample differences, where studies that included healthy participants tended to find a negative relationship between ePVS and perfusion, while most studies involving patient samples did not. While only few studies separately assessed ePVS regions, all studies investigating CSO ePVS found no significant association between CSO ePVS and perfusion, while BG ePVS only had a significant relationship with perfusion in a healthy cohort.

### ePVS and free water

Five papers investigated the relationship between ePVS and extracellular free water, quantified by T2-weighted MRI and diffusion MRI respectively.

Huang et al. [41] found that deep white matter ePVS volumes were significantly positively associated with free water in a healthy cohort, even when stratifying the sample into low WMH (below median) and high WMH (above median) loads. Mediation analyses in this study revealed that free water fully mediated the positive relationship between ePVS and WMH in those with a high-WMH load. In a further study of the same community cohort and additionally comparative CSVD cohort, Huang et al. [66] showed that the positive association between ePVS and free water exists in both healthy participants and in CSVD patients. Lan et al. [67] further supported this association in CSVD patients, demonstrating that a higher number of BG ePVS correlated with higher free water volumes.

Jiaerken et al. [68] investigated free water levels within the surrounds of white matter ePVS across three groups: healthy younger adults, healthy elderly and those with CSVD. They found significantly higher free water levels within and immediately around (0–2 mm) ePVS compared to non-ePVS reference voxels in all groups. People with CSVD showed the greatest difference between free water levels in ePVS and non-ePVS reference voxels, while healthy younger adults showed the least difference. Across all groups, free water levels decreased proportionally with increasing distance from ePVS, but free water levels within ePVS were only strongly correlated with free water levels within normal-appearing white matter in the healthy groups.

Zotin et al. (gray literature) [69] found an association between CSO ePVS and free water in a cohort of probable cerebral amyloid angiopathy patients.

Overall, the studies consistently found a positive association between ePVS and free water across both healthy and patient samples. However, the levels of free water inside of ePVS appear to be significantly higher in those with CSVD compared to healthy persons.

Regarding the relationship between ePVS and WMH, studies consistently found a positive association between ePVS and WMH in healthy samples [70–83], as well as in individuals with cognitive impairment or stroke [84–89]. BG ePVS was consistently positively associated with WMH, while the relationship with CSO ePVS was less discernible, with studies reporting negative or null associations [90–96].

### BBB permeability and perfusion

Eight papers investigated the relationship between BBB permeability and cerebral perfusion using various DCE MRI, ASL and dynamic susceptibility contrast techniques.

Chen B. et al. (gray literature) [97] found that in patients with asymptomatic carotid artery stenosis, areas with increased BBB permeability corresponded to regions of hypoperfusion and brain atrophy.

van de Haar et al. [98] found a significant negative correlation between pseudo-continuous ASL CBF and BBB permeability in patients with mild cognitive impairment or Alzheimer’s disease, but not in a group of healthy participants. Additionally, the fraction of leaking voxels on DCE MRI was not significantly associated with CBF in either the healthy or mild cognitive impairment/Alzheimer’s disease groups.

Wong et al. [99] demonstrated a relationship between BBB permeability and CBF (measured by dynamic susceptibility contrast MRI) in those with CSVD, where both the volume of leakage and contrast influx rate were associated with lower CBF in both normal-appearing white matter and in areas of WMH. This strength of this coupling was reportedly stronger with closer proximity to regions of WMH.

Chi et al. [100] investigated the association between BBB permeability (and volume of contrast in the extracellular space) and DCE CBF in patients with systemic lupus erythematosus. Both BBB permeability and extracellular contrast volume were found to be significantly positively associated with CBF. However, in healthy subjects, no associations between either measure and CBF were found.

Similarly, Varatharaj et al. [101] reported that in healthy participants, cerebral blood volume but not CBF was associated with BBB permeability.

Haselhorst et al. [102] found that patients with multiple sclerosis showed increased regional cerebral blood volume in strongly enhancing gadolinium plaques relative to normal-appearing white matter.

Wuerfel et al. [103] found increased CBF signal when gadolinium signal enhancement plaques were identified, including in pre-relapse remittent multiple sclerosis lesion regions up to 3 weeks prior to evidence of enhancement.

Wengler et al. [104] investigated the relationship between the BBB trans-capillary water extraction fraction and CBF using pseudo-diffusion pseudo-continuous ASL. BBB extraction fraction was negatively associated with CBF, indicating that greater blood flow was associated with lower permeability. Additionally, water permeability surface area product showed a high correlation with CBF.

Overall, studies that investigated inter-relationships between BBB permeability and perfusion using DCE MRI and ASL MRI tended to find negative associations between BBB permeability and blood flow, although results from studies using other perfusion methodologies are mixed. There were mixed findings within group types, but predominantly, studies investigating healthy groups tended to find no significant relationships between BBB permeability and perfusion. In contrast, studies investigating relapse-remitting multiple sclerosis and systemic lupus erythematosus found a positive association, while a negative relationship was found in mild cognitive impairment/Alzheimer’s disease and CSVD groups.

### BBB permeability and free water

Only one study investigating the association between BBB permeability and extracellular free water was identified. Hillmer et al. [105] investigated a clinical sample consisting of patients with Alzheimer’s disease, vascular cognitive impairment and dementia or leukoaraiosis patients. In this sample, BBB permeability was positively correlated with mean free water levels. Regarding literature investigating WMH, studies generally suggested a positive relationship between BBB permeability and WMH [105–110]. One longitudinal study showed that increased BBB permeability preceded some WMH lesions [111].

### Perfusion and free water

While there were no identified studies that investigated the association between perfusion measures and free water, there were a substantial number of papers investigating perfusion and WMH. Results generally suggested a negative relationship, where lower perfusion was associated with a greater volume of WMH [112–141].

## Discussion

This review identified a growing body of work using MRI measures to investigate links between different aspects of NVU in health, aging and neurodegenerative pathology. We identify differences in the presence, strength, and region of inter-relationships in populations with and without neurodegenerative pathology and highlight two putative clusters of NVU subcomponents that appear to be strongly inter-dependent: (1) BBB permeability, perfusion and BG ePVS (the “vascular” cluster), and (2) ePVS, free water and WMH (the “fluid” cluster).

### The presence, strength and region of NVU subcomponent inter-relationships

Interdependencies between some aspects of NVU structure and function may be modified by neurodegenerative pathology. In particular, a greater number of ePVS, especially BG ePVS, is associated with higher BBB permeability and lower perfusion in non-clinical populations. However, these relationships are reduced or abolished in individuals with stroke, CSVD or pathology featuring cognitive impairment. In contrast, studies investigating BBB permeability and perfusion found that there is typically no inter-relationship between these measures in the healthy population, but negative or positive associations were variably found in multiple sclerosis, mild cognitive impairment/Alzheimer’s disease and CSVD. These observations suggest that some inter-relationships between MRI measures of NVU function are indicative of the normative function of the NVU as an integrated system, while others may be indicative of cascading pathological processes or NVU breakdown. As many neurodegenerative diseases feature a pathological cascade of metabolic and structural changes in the brain and vasculature, it is plausible that changes within and between elements of the NVU may reflect a core, but potentially subtle, mechanistic underpinning of this pathology. Future research investigating NVU inter-relationships throughout a context of ‘healthy’ aging and in pathology may characterise the presence or de-coupling of some inter-relationships as intricate early markers of neurodegenerative pathology.

Notably, not all inter-relationships covered in the scope of this review differed in the presence of pathology. For example, ePVS and free water were consistently positively associated in both healthy and patient cohorts. Interestingly, though, free water was shown to mediate the relationship between ePVS and WMH, but only when the WMH load was considered ‘high’. It is therefore possible that the association between free water and ePVS increases in strength with pathological processes or breakdown of the NVU, although longitudinal research is required to confirm this hypothesis.

Although the majority of literature covered in this review quantified each NVU measure in average across the whole brain, when ePVS were investigated in the BG ePVS and in the CSO ePVS separately, different associations with other measures of NVU function were evident. Specifically, while BG ePVS tended to have associations with other MRI measures (perfusion, BBB permeability) in both healthy samples and in those with vascular-related conditions such as CSVD or stroke, CSO ePVS associations with other MRI measures (free water) were only evident in those with probable cerebral amyloid angiopathy. Although literature investigating CSO ePVS was limited in the studies included in this review, this finding is consistent with other research suggesting that BG ePVS are more prevalent in diseases with a vascular component or origin, such as atherosclerosis [142] or vascular parkinsonism [143]. Conversely, CSO ePVS are associated with the progression of diseases with increased amyloid deposition, including Alzheimer’s disease [144] and cerebral amyloid angiopathy [91, 142]. It is therefore possible that quantifying BG and CSO ePVS separately may be useful in differentiating vascular-based pathology from other neurodegenerative pathology.

Besides ePVS, most of the reviewed literature quantified MRI outcomes globally. Despite this, it is possible that inter-relationships may change in presence or strength within different brain areas to reflect underlying pathology, particularly in diseases that are characterised by region-specific degeneration (such hippocampal atrophy in Alzheimer’s disease) or which feature localised lesions (such as multiple sclerosis). This review highlights the lack of knowledge in this area, and future research is required to explore these relationships across brain regions and different pathologies.

### BG ePVS, perfusion and BBB permeability: the vascular cluster

Across the reviewed literature, there appears to be relatively consistent evidence of strong links between increased CBF, reduced BBB integrity, and increased prevalence of BG ePVS in pathological conditions. With respect to BBB permeability and ePVS, increased BBB permeability may also result in an increase of molecules passing into the perivascular space which could obstruct the drainage passageway and contribute to MR visible enlargement. Furthermore, fluid exchange through the BBB and AQP4 channels is thought to facilitate cerebrospinal fluid flow within perivascular spaces [145], suggesting that both of these components of the NVU may contribute to MR-visible ePVS. Importantly, a relationship between ePVS and BBB permeability was not found in non-pathological cohorts in this review, suggesting that pathology or perhaps more severe NVU dysfunction may underlie a possible coupling of these features. However, this lack of association may also be due to the challenges of measuring the BBB in vivo, the relative nascence of this research and, subsequently, the limited literature. Similarly, perfusion and BBB permeability also did not have a significant inter-relationship within healthy cohorts, but indices of these features are correlated in those with CSVD, carotid artery stenosis and mild cognitive impairment/Alzheimer’s disease. Collectively, these findings may suggest that the presence of correlations between BBB permeability, perfusion and/or BG ePVS may be indicative of NVU dysfunction beyond what is typical for a healthy population, perhaps even providing a composite novel marker of CSVD and neurodegenerative disease. Although this review did not find any articles investigating the relationship between ePVS and BBB permeability in CSVD, it is worth investigating whether an inter-relationship emerges with CSVD or proportionately to CSVD severity, given there is evidence of a relationship between BBB permeability and perfusion in CSVD.

### ePVS, free water and WMH: the fluid cluster

Clustering of NVU dysfunction measures also occurred between ePVS, free water and WMH, which may reflect dysfunction of the glymphatic or fluid regulation aspects of NVU function. Both ePVS and free water are measures considered to be indicative of the dysfunction of interstitial fluid exchange or the stagnation of perivascular drainage [31, 32, 39–41]. Concordantly, studies included in this review were unanimous in supporting a positive inter-relationship between ePVS and extracellular free water in both healthy and CSVD subjects. It is possible that free water is also indicative of reduced microstructural integrity at a pre-WMH stage, as normal-appearing white matter conversion to WMH is predicted by increased baseline free water levels [47].

Interestingly, greater ePVS is also predictive of an increase in WMH, but WMH is not predictive of increased ePVS [76]. This uni-directional association helps contextualise the relationship between ePVS and free water. Perhaps the dysfunction of perivascular space contributes to poorer glymphatic drainage of free water in interstitial fluid in the parenchyma, which in turn may contribute to WMH. As such, the inter-relationship between both ePVS and free water may better reflect of glymphatic dysfunction than either measure alone, presenting the opportunity to identify those at risk of eventual neurodegeneration before WMH or other atrophy markers appear. Indeed, for both healthy and CSVD subjects, free water levels increased with proximity to ePVS voxels, but this relationship was stronger in those with CSVD [68]. One explanation for this inter-relationship might lie in the interpretation of what ePVS represent. For those who are healthy, enlargement of the perivascular space may help facilitate CSF drainage, as suggested by an increase in CSF flow during sleep [146–148], resulting in greater fluid flow within the ePVS and less fluid diffusion in the surrounding brain tissue [68]. Conversely, in CSVD or other pathologies, ePVS may reflect glymphatic impairments, stagnation of CSF within the NVU, and great diffusion of water molecules into the brain, ultimately representing failure of homeostatic compensation. This idea is further supported by evidence that WMH, which is predicted by free water accumulation [47], preferentially form around ePVS [41]. Finally, free water seems to fully mediate the relationship between WMH and ePVS, but only in those with high WMH [41]. Thus, the inter-relationship between free water and ePVS may be useful in distinguishing normal glymphatic variability from change reflective of CSVD risk or pathology.

### Limitations

Although we have been able to draw several novel insights regarding the structure of the NVU in both the healthy brain and in disease, our ability to definitively address the aims of this review is limited by several aspects of currently available literature. In particular, although a number of valuable cross-sectional studies have been undertaken, there have been no longitudinal investigations of NVU inter-relationships. As such, characterisation of the evolving nature of NVU dysfunction with disease progression requires further research.

It is also important to note that there is an unbalanced availability of research investigating different MRI measure associations. Associations more frequently researched may mask or over-extenuate different clusters of NVU coupling. For example, there were fewer studies investigating the associations between ePVS and BBB permeability than ePVS and perfusion, limiting our confidence in comparing and clustering these association pairs.

While there was reasonable sample diversity within measure pairs, healthy cohorts tended to have a larger sample size than cohorts of specific vascular or neurological disease. Given that this review focused on the statistical significance of investigated associations that are heavily influenced by sample and effect size, the insights drawn within this review may be subject to this imbalance. Furthermore, given that several studies identified in this review had relatively small sample sizes, interpreting statistical significance alone as evidence of a real association may have masked the ability to infer more subtle relationships that emerge with larger population studies.

There is growing evidence to suggest that some medications that are widely used by the general population affect neurovascular functioning and health, such as anti-hypertensives and statins [149]. Notably, we found that the reviewed studies inconsistently described medication use within their research cohorts, thus limiting our confidence to interpret findings as independent from their potential effects. While some studies incorporated inclusion/exclusion criteria regarding specific categories of medications or consistency of medication use, most studies did not report or adjust for medication use in statistical analyses. The potential confounding influence of medication effects on the observed interdependencies between NVU components thus remains unclear. Future research should consider medication use as a possible extraneous or moderating factor when investigating NVU integrity and function.

In this review, we have identified and focused on MRI measures considered to be representative of NVU integrity or dysfunction. However, the interpretation of these outcomes is dependent upon the theoretical assumptions of their underlying biophysiological mechanisms. We have discussed the impairment of glymphatic system or interstitial fluid exchange as an explanation for MR-visible enlargement of the perivascular space and the accumulation of extracellular free water. However, the glymphatic hypothesis has been contended in this context, where solute transport through the NVU into the parenchyma may be more limited than presumed [150, 151].

Interpretation limitations also arise from the methodology frequently used within identified studies. For instance, the Patlak pharmacokinetic equation for quantifying BBB permeability from DCE MRI is dependent upon the delivery of a contrast via the blood stream. It can be expected that a positive relationship between blood flow and BBB permeability exists through method of quantification alone, as k^trans^ is highly dependent on plasma flow or CBF. Importantly, studies identified in this review which used DCE MRI to quantify *both* BBB permeability and CBF tended to find a non-significant or positive inter-relationship between the measures. In contrast, studies that used DCE MRI for BBB permeability and instead used ASL for CBF quantification tended to find a non-significant or negative association. Future research is encouraged to use methodologically independent measures when assessing the inter-relationships within the NVU.

In addition to methodological technique, the quantification approach may also impact the interpretation of inter-relationship findings. One study found variability in associations between ePVS and WMH across differing quantification methods. Although BG ePVS and WMH had a consistent positive relationship, non-BG ePVS were only associated with WMH volume when ePVS was quantified as a manual count (rather than convolutional neural network segmentation volume) [79]. Although there is insufficient literature comparing quantification approach associations, it is possible some quantification approaches are more sensitive to subtle NVU change than others. It is also possible that the different quantification methods reflect different concepts, where in this case higher ePVS manual count may reflect more profuse and consistent vascular dysfunction while ePVS volume may highlight specific local areas of NVU structure disruption. Notably, MRI sequences are currently being developed to measure the water exchange rate through the NVU, including the works from Shao, Wang, Wengler and others [104, 152–154]. These very novel sequences may help verify current interpretations of fluid flow in the NVU, including possibly theoretically differentiating ePVS manual count and ePVS volume. This measure of ‘water exchange’, which represents the diffusion of water molecules from within the BBB to the interstitial space in the parenchyma, will provide opportunity to better understand the role of water transport, and interstitial fluid exchange, in both a healthy and vascular-impaired brain.

Lastly, several conditions or brain features that were outside the scope of this review may still be relevant to neurovascular health and NVU component interdependencies. For example, hydrocephalus or ventricle enlargement, cerebral microbleeds and lacunae are all identifiable or measurable in the general population and may modify or contribute to neurovascular health and thus sub-structural interdependencies [155, 156].

## Conclusion

The NVU is a complex system formed by multiple subcomponents that can be individually assessed in vivo using diverse MRI measures. A growing body of literature has used multi-modal imaging approaches to investigate the varying inter-relationships between these subcomponents in non-clinical, aging, and neurodegenerative disease populations. While this literature is relatively nascent, consistent and potentially differential inter-relationships are emerging that may have relevance to pathological progression or advanced aging. The findings suggest that MRI NVU dysfunction measures may cluster in consistent ways across different pathological states, potentially serving as detailed and differential indicators of underlying pathology and possible novel markers for early detection and progressive tracking of neurodegenerative disease. In particular, we highlighted two potential clusters of NVU subcomponent interdependencies, orientated around ‘vascular’ dysfunction (BBB permeability, perfusion and BG ePVS) and ‘fluid’ transport dysfunction (ePVS, free water and WMH). Future research is required to further characterise these inter-relationships in aging and in different pathologies. Focused statistical analyses, such as a principal components analysis, may provide empirical evidence for the differential capacity of NVU inter-relationships. Additionally, by investigating these associations longitudinally, it will also be possible to determine whether there is a cascading nature to NVU dysfunction within these clusters that may co-occur with specific pathological mechanisms.

### Supplementary Information

**Additional file 1: Summary of WMH study samples, MRI methodologies and relationship investigated.** Included studies investigating the relationship between key MR markers and WMH, their study design, and a qualitative summary of their findings. Marker associations are summarised as either ‘positive’, ‘negative’, ‘unspecified’ (analysis does not detail relationship direction) or ‘non-significant’ (did not meet statistical significance). Strength and statistical significance of relationship reported where available. Models adjusted for age and sex, or minimal models reported. Age is reported from baseline statistics in longitudinal studies.

## Data Availability

Data sharing is not applicable to this article as no datasets were generated or analysed during the current study.

## Abbreviations

ASL: Arterial spin labelling
AQP4: Aquaporin-4
BBB: Blood–brain barrier
BG: Basal ganglia
CBF: Cerebral blood flow
CSO: Centrum semiovale
CSVD: Cerebral small vessel disease
DCE: Dynamic contrast enhanced
ePVS: Enlarged perivascular spaces
NVU: Neurovascular unit
WMH: White matter hyperintensities
