# Supplementary material for: Imaging the neurovascular unit in health and neurodegeneration: a scoping review of interdependencies between MRI measures
Source: Fluids Barriers CNS. 2023 Dec 21;20:97. doi: 10.1186/s12987-023-00499-0 (PMC10734164; doi:10.1186/s12987-023-00499-0)
Supplement: Supplementary file 1 — Additional file 1: Summary of WMH study samples, MRI methodologies and relationship investigated. Included studies investigating the relationship between key MR markers and WMH, their study design, and a qualitative summary of their findings. Marker associations are summarised as either ‘positive’, ‘negative’, ‘unspecified’ (analysis does not detail relationship direction) or ‘non-significant’ (did not meet statistical significance). Strength and statistical significance of relationship reported where available. Models adjusted for age and sex, or minimal models reported. Age is reported from baseline statistics in longitudinal studies. [file 12987_2023_499_MOESM1_ESM.docx]

Imaging the neurovascular unit in health and neurodegeneration: a scoping review of interdependencies between MRI measures

Supplementary Information

Ella Rowsthorn^a,b^, William Pham^a^, Mohammad-Reza Nazem-Zadeh^a^, Meng Law^a,c,d^, Matthew P. Pase^b,e^*, Ian H. Harding^a,f^*^#^

*These authors contributed equally.
^#^Corresponding author.

1. Department of Neuroscience, Central Clinical School, Monash University, 99 Commercial Road, Melbourne, VIC 3004, Australia
2. Turner Institute for Brain and Mental Health & School of Psychological Sciences, Monash University, 18 Innovation Walk, Clayton, VIC 3168, Australia
3. Department of Radiology, Alfred Health, 99 Commercial Road, Melbourne, VIC 3004, Australia
4. Department of Electrical and Computer Systems Engineering, Monash University, 14 Alliance Lane, Clayton, VIC 3168, Australia
5. Harvard T.H. Chan School of Public Health, 677 Huntington Avenue, Boston, MA 02115, USA
6. Monash Biomedical Imaging, Monash University, 762-772 Blackburn Road, Clayton, VIC 3168, Australia

Declarations of interest: none.

**Correspondence:**

Dr Ian H. Harding

Department of Neuroscience

Monash University

99 Commercial Road
Melbourne VIC 3004
Australia

E: [ian.harding@monash.edu](mailto:ian.harding@monash.edu)

T: +61-3-9905-9283

**Database Search Strategies**

**Ovid Medline (R) All Search**

Topic 1:

(ePVS or PVS or perivascular-space* or Virchow-Robin).ab,kf,ti.

Topic 2:

(*Blood Brain Barrier/ or (BBB or blood-brain-barrier or blood-brain or BCSFB).ab,kf,ti.) and (permeab* or leak* or exchang* or integrity*).ab,kf,ti.

Topic 3:

Cerebrovascular Circulation/ or (CBF or rCBF or CBV or rCBV or cerebral-blood-volume or cerebral-perfusion or brain-perfusion or perfusion-neuro or cerebral-hemody* or brain-hemody*).ab,kf,ti.

Topic 4:

(free-water or isotropic-volume-fraction or isotropic-diffusion or ISOVF or white-matter-hyperinten* or WMH).ab,kf,ti.

Final Search: (limited to English)

((1 and (2 or 3 or 4)) or (2 and (3 or 4)) or (3 and 4)) not review.pt. not (Animal/ not (Animal/ and Human/))

**Ovid Embase Search (Includes Grey Literature)**

Topic 1:

(ePVS or PVS or perivascular-space* or Virchow-Robin).ab,kf,ti.

Topic 2:

(*Blood Brain Barrier/ or (BBB or blood-brain-barrier or blood-brain or BCSFB).ab,kf,ti.) and (permeab* or leak* or exchang* or integrity*).ab,kf,ti.

Topic 3:

Cerebrovascular Circulation/ or (CBF or rCBF or CBV or rCBV or cerebral-blood-volume or cerebral-perfusion or brain-perfusion or perfusion-neuro or cerebral-hemody* or brain-hemody*).ab,kf,ti.

Topic 4:

(free-water or isotropic-volume-fraction or isotropic-diffusion or ISOVF or white-matter-hyperinten* or WMH).ab,kf,ti.

Final Search: (limited to English)

((1 and (2 or 3 or 4)) or (2 and (3 or 4)) or (3 and 4)) not review.pt. not ((exp animal/ or exp invertebrate/ or nonhuman/ or animal experiment/ or animal tissue/ or animal model/ or exp plant/ or exp fungus/) not (exp human/ or human tissue/))

**Web of Science Search**

Topic 1:

TS=(ePVS or PVS or perivascular-space* or Virchow-Robin)

Topic 2:

TS=(((permeab* or leak* or exchang* or integrity *) NEAR/1 BBB) or ((permeab* or leak* or exchang* or integrity *) NEAR/1 blood-brain-barrier) or ((permeab* or leak* or exchang* or integrity*) NEAR/1 blood-brain))

Topic 3:

TS=(cerebral-blood-flow or CBF or rCBF or cerebral-blood-volume or CBV or rCBV or cerebral-perfusion or brain-perfusion or perfusion-neuro* or cerebral-hemody* or brain-hemody*)

Topic 4:

TS=(free-water or isotropic-volume-fraction or isotropic-diffusion or ISOVF or white-matter-hyperinten* or WMH)

Final Search: (limited to English – Language and exclude Review Article – Document Type)

(#1 and (#2 or #3 or #4)) or (#2 and (#3 or #4)) or (#3 and #4)

***Supplementary Table 1***

| **Author (Year)** | **Sample (*N*)*** | **Longitudinal** | **Age, M ± *SD*** | **MRI Method^#** | | **WMH Inter-relationship Summary** | |
| --- | --- | --- | --- | --- | --- | --- | --- |
| ***ePVS x WMH ePVS WMH Association Summary*** | | | | | | | |
| Arba et al. (2018) | Stroke/TIA (N=407) |  | 65 **±** 13 | T2 visual count grade | T2 FLAIR Fazekas | BG and CSO ePVS vs WMH, for both PWMH and DWMH (*r*=0.39-0.61, p<.001).  Total ePVS vs WMH, for DWMH (β=0.31, *p*<.001). Total ePVS vs WMH, for PWMH (β=0.13, *p*=.087) | Positive  Positive  Non-significant |
| Aribisala et al. (2014) | Healthy (N=634) |  | 73 **±** 1 | 1.5T T2 visual count grade (hippocampus/BG/CSO) | 1.5T T2 FLAIR volume and Fazekas | ePVS vs WMH (β=0.47, *p*<.0001), all ePVS regions correlated with WMH (*r*=0.11-0.32, *p*<.001). | Positive |
| Ballerini et al. (2020) | Healthy (N=533) |  | 73 **±** 1 | 1.5T T2 volume, visual count and grade | 1.5T T2 FLAIR volume and Fazekas | ePVS vs WMH (β=0.14-0.66, *p*<.05). ePVS mean size had the strongest association (β=0.43-0.66, *p*<.05) | Positive |
| Barnes et al. (2022) | Healthy (N=29) | Yes | 72 **±** 1 | 1.5T T2 segmentation | 1.5T T2 & T2 FLAIR segmentation | ePVS vs WMH, for baseline ePVS proximity and WMH progression (OR=2.00-2.44, *p*=0.17-0.59) | Non-significant |
| Bouvy et al. (2016) | Healthy (N=50) |  | 63 **±** 9 | 7T T2 visual count | 1.5T/3T T2 FLAIR volume | BG ePVS vs WMH (*B*=1.3, *p*=.02)  CSO ePVS vs WMH (*B*=5.6, *p*=.07) | Positive  Non-significant |
| Charidimou et al. (2016) | CAA ICH (n=319) Hypertensive Arteriopathy ICH (n=137) |  | 74 (95% CI=72.7-75.1) 67 (95% CI=64.8-69.5) | 1.5T T2 visual count | 1.5T T2 FLAIR patterns | BG ePVS vs WMH, for peri-BG pattern (OR=8.81, *p*<.001).  CSO ePVS vs WMH, for peri-BG pattern (OR=0.56, *p*=.154)  CSO ePVS vs WMH, for multiple subcortical WMH spots (OR=2.43, *p*<.0001).  BG ePVS vs WMH, for multiple subcortical WMH spots (OR=0.64, *p*=.418). | Positive  Non-significant  Positive  Non-significant |
| Charidimou et al. (2017) | CAA ICH (n=315) Hypertensive Arteriopathy ICH (n=137) |  | 74 (95% CI=72.7-75.1) 67 (95% CI=64.8-69.5) | 1.5T T2 visual count grade | 1.5T T2 FLAIR volume | BG ePVS vs WMH, comparing low (0-2) to high (3-4) grade ePVS (*p*=.001)  CSO ePVS vs WMH, comparing low to high grade ePVS (*p*=.237) | Positive   Non-significant |
| De Guio et al. (2018) | CADASIL (n=11) |  | Not reported | 7T T2* location | 7T T2* location | ePVS vs WMH, ePVS did not share location with WMH in 7T scans. | Non-significant |
| Del Brutto et al. (2022) | Healthy (N=263) | Yes | 66 **±** 6 | 1.5T T2 visual count grade | 1.5T T2/T2 FLAIR | ePVS vs WMH, WMH progression predicted BG-ePVS progression (*OR*=4.45, *p*<.05). | Unspecified |
| Ding et al. (2017) | Healthy (N=2612) | Yes | 75 **±** 5 | 1.5T T2 visual count (BG/ arterial white matter) | 1.5T T2 FLAIR volume | ePVS vs WMH, when comparing baseline no/mild/severe ePVS to WMH progression (β=0.03-0.06, *p*<.001) | Positive |
| Duperron et al. (2018) | Healthy (N=1562) |  | 73 **±** 4 | 1.5T T2 visual count grade (BG/white matter/hippocampus) | 1.5T T2 volume | ePVS vs WMH, for global (*rho*=0.27, *p*<.0001)  ePVS vs WMH, for white matter/BG ePVS and deep/periventricular WMH (*rho*=0.08-0.33, *p*<.0001) | Unspecified  Unspecified |
| Evans et al. (2022) | Healthy (N=39,976) |  | 65 (range 20-96) | 1.5T/3T T2 visual or neural network count (BG/CSO/hippocampus /mesencephalon) | 1.5T/3T T1/T2 FLAIR | ePVS vs WMH, hippocampus (OR=1.07), BG (OR=1.13) and CSO (OR=1.10) (corrected *p*<.05 for all). ePVS vs WMH, mesencephalon (OR=1.03, corrected *p*>.05). | Unspecified  Non-significant |
| Gertje et al. (2021) | Healthy (n=499) MCI (n=240) |  | 72 **±** 6  71 **±** 5 | T1/T2 FLAIR visual count (BG/CSO/ hippocampus) | T2 FLAIR Fazekas and volume | ePVS vs WMH volume, for CSO (β=0.232, *p*<.001), BG (β=0.251, *p*=.001) and hippocampal (β=0.203, *p*<.001) ePVS. Similar results for ePVS vs Fazekas. | Positive |
| Gyanwali et al. (2019) | Healthy/MCI (n=375) Healthy/MCI (n=583) |  | med=73, IQR=12  med=70, IQR=11 | T1/T2 visual count (BG/CSO/hippocampus /mesencephalon) | T1/T2 volume | Total ePVS vs WMH (rate ratio=1.00) BG ePVS vs severe WMH, in cognitive impaired only (rate ratio=1.45, *p*=.012)  CSO ePVS vs severe WMH, in healthy only (rate ratio=0.76, *p*<.001) | Non-significant  Positive  Negative |
| Hakim et al. (2021; grey literature) | Ischemic Stroke (N=254) |  | 69 **±** 15 | Not reported | Not reported | ePVS vs WMH (*stats not reported*). | Non-significant |
| Huo et al. (2022) | Confluent WMH (n=32) Healthy (n=16) |  | 44 **±** 6  43 **±** 6 | 7T T2 visual count grade | 7T T2 FLAIR count | CSO ePVS vs deep WMH, when comparing >5 WMHs to without WMH (Z=-2.794, *p*=.014). BG ePVS vs deep WMH, when comparing >5 WMHs to without WMH (Z=-1.791, *p*=.381).  ePVS vs deep WMH, when comparing <5 WMH to without (Z=0 - -0.314, *p*=.809-1.00).  Positive ePVS vs WMH, when comparing ePVS dilation and number in NAWM to deep WMH (Z=-7.184, *p*<.001). 79.59% of deep WMH were spatially connected to PVS tubes. | Positive   Non-significant  Non-significant  Positive |
| Hurford et al. (2014) | TIA/Stroke (N=246) |  | 62 **±** 17 | 1.5T T2 visual count grade | 1.5T T2 FLAIR Fazekas | ePVS vs WMH, for CSO and BG (OR=1.12-1.38, *p*<.0001) | Positive |
| Lau et al. (2017) | TIA/Stroke (n=1028) Ischemic Stroke (n=974) |  | 68 **±** 14  69 **±** 12 | 1.5T/3T T2 visual count | 1.5T/3T T2 FLAIR Fazekas | BG ePVS vs WMH, for ePVS>20 and PVWMH and subcortical WMH (OR=1.44-2.01, *p*<.0001). CSO ePVS vs WMH, for ePVS>20 and subcortical WMH (OR=1.28, *p*=.003)  CSO ePVS vs WMH, for PVWMH (OR=0.90, *p*=.26). | Positive  Positive  Non-significant |
| Laveskog et al. (2018) | Healthy (N=530) |  | 71 **±** 12 | 1.5T T2 visual count grade | 1.5T T2 FLAIR scale | ePVS vs WMH, for both count and size of ePVS (*p*<.05). | Positive |
| Li Y et al. (2022) | Healthy (N=325) | Yes | 70 **±** 8 | 1.5T T1/T2 visual count | 1.5T T1/T2 FLAIR score | ePVS vs WMH, basline ePVS with global and lobar WMH increase (β=0.05-0.07, *p*<.001). ePVS vs WMH, baseline WMH associated with ePVS increase (β=0.03-0.08, *p>*.05). | Positive   Non-significant |
| Libecap et al. (2022) | Healthy/MCI (N=106) |  | 70 **±** 6 | T1/T2 FLAIR visual count (BG/CSO/midbrain/ hippocampus) | T2 FLAIR volume | ePVS vs WMH, with total ePVS (β=0.245, *p*=.01), BG ePVS (β=0.324, *p*<.001) and CSO ePVS (β=0.206, *p*=.027). ePVS vs WMH, with midbrain ePVS (*p*=.117) and hippocampal ePVS (*p*=.860). | Positive  Non-significant |
| Lin et al. (2021) | CMBs and Dementia (n=46) CMBs (n=12) |  | 75 (range 72-82)  72 (range 64-83) | 1.5T visual count grade | 1.5T T2/T2 FLAIR Age-Related White Matter Change scale | ePVS vs WMH, for either BG or CSO ePVS in total, infratentorial, BG or subcortical WMH regions (*r*=-0.179–0.068, all *p*>.05). | Non-significant |
| Martinez-Ramirez et al. (2013) | MCI/AD (N=89) |  | 73 **±** 10 | T1/T2 visual count grade (BG only) | T2 FLAIR | ePVS vs WMH, when comparing low/high BG ePVS groups (*p*=.02) ePVS vs WMH, when comparing low/high white matter ePVS groups (*p*=.43). | Positive  Non-significant |
| Paradise et al. (2020) | Healthy (N=414) |  | 80 **±** 5 | T1/T2 visual count | T2 FLAIR volume | BG ePVS vs WMH (*r*=0.26, *p*<.01). CSO ePVS vs WMH (*r*=.0.06, *p*>.05). | Positive  Non-significant |
| Ramirez et al. (2015) | AD (n=203) Healthy (n=94) |  | 73 **±** 9  70 **±** 8 | 1.5T T1/T2 visual count and volume | 1.5T T1/T2 count | AD: ePVS vs WMH (*r*=0.3, *p*<.01). Healthy: ePVS vs WMH (*significance not reported*). | Positive  Non-significant |
| Rodriguez Lara et al. (2023) | Healthy (N=2454) |  | 54 **±** 12 | 1T-3T T2 visual count grade | 1T-3T T2 FLAIR volume | ePVS vs WMH, for BG (β=0.55, *p*<.001), CSO ePVS (β=0.58, *p*<.001) or mixed region ePVS (β=0.88, *p*<.001). | Positive |
| Sahin et al. (2015) | ICAS (N=29) |  | 69 **±** 9 | 1.5T T1/T2/FLAIR | 1.5T T1/T2/FLAIR | ePVS vs WMH, for BG ePVS (*significance not reported*). ePVS vs WMH, for CSO ePVS (*significance not reported*). | Unspecified   Non-significant |
| Shams et al. (2017) | Memory Impairment (N=1504) |  | 63 **±** 10 | 1.5T/3T T2 visual grade | 1.5T/3T T2 FLAIR Fazekas | ePVS vs WMH, for moderate/severe WMH and BG (OR=8.3, *p*<.0001) and CSO ePVS (OR=3.7, *p*<.0001). | Positive |
| Shen et al. (2020) | ICAS (N=202) |  | 62 **±** 12 | T2 visual count grade | T2 FLAIR Fazekas | ePVS vs WMH, for BG (*G*=.485, *p*<.001) and white matter ePVS (*G*=.424, *p*<.001) | Positive |
| Thanprasertsuk et al. (2014) | Evidence of CAA (n=59) CAA (n=41)  Non-CAA (n=18) |  | 74 **±** 9  75 **±** 8  72 **±** 10 | 1.5T/3T T2 visual count | 1.5T/3T volume and anterior position center of WMH | ePVS vs WMH, WMH volume different across ePVS categories (*p*=0.42). ePVS vs WMH, where distribution of WMH was significantly different across ePVS categories (*p*=.001). | Unspecified  Unspecified |
| Tommasino et al. (2022; grey literature) | Post SARS CoV-2 Infection (N=107) |  | 44 | Not reported | Not reported | ePVS vs WMH (*R*=0.35, *p*=.0171). | Unspecified |
| Wang ML et al. (2021) | Healthy (N=106) |  | 75 **±** 6 | T1/T2 visual count grade | T2 FLAIR Fazekas | BG ePVS vs WMH, when comparing low grade (1) to high (2-4) ePVS for PWMH (*p*=.010) and DWMH (*p*=.004). CSO ePVS vs WMH, when comparing low grade (1) to high (2-4) ePVS for PWMH (*p*=.526) and DWMH (*p*=.052). | Positive  Non-significant |
| Wang S et al. (2021) | Healthy (N=161) |  | 60 **±** 7 | T2 visual count grade and neural network volume (BG/WM=non-BG non-peri-ventricular) | T2 FLAIR Fazekas and volume | BG ePVS vs WMH, for all ePVS quantification methods with WMH volume (β=0.188-0.268, *p*=.016-.001) and Fazekas (β=0.146-0.265, *p*=.085-.001). WM ePVS vs WMH volume, for ePVS count only (β=0.250, *p*=.001).  WM ePVS vs WMH volume/WMH Fazekas, some quantification methods. | Positive  Positive  Non-significant |
| Wang X et al. (2016) | Lacunar or mild cortical stroke (N=100) |  | 69 (range 37-92) | 1.5T T1/T2 visual count grade | 1.5T T2 FLAIR Fazekas and volume | BG ePVS vs WMH, for Fazekas (B=1.92-2.20, *p*<.001) and volume (B=0.065-0.096, *p*<.001). | Positive |
| Wang XY et al. (2022) | ICAS (N=516) |  | 59 **±** 20 | T2 visual count grade | T2 FLAIR Fazekas | BG ePVS vs WMH, for severe WMH (pWMH grade 3+ or deep WMH grade 2-3) (*R*=0.478, *p*<.05). | Unspecified |
| Xia et al. (2020) | Healthy (N=191) | Yes | median = 68, IQR = 63-73 | 1.5T/3T T1/T2* visual count grade | 1.5T/3T T2 FLAIR volume | ePVS vs WMH, for WMH predicting ePVS progression (β=0.02, 95% CI =-0.05-0.09, *p*>.05). ePVS vs WMH, for ePVS predicting WMH progression (β=2.89, 95% CI =1.14-7.32, *p*<.05). | Non-significant  Positive |
| Yakushiji et al. (2014) | Healthy (N=1575) |  | 57 **±** 10 | 1.5T T2 visual count grade | 1.5T T2 FLAIR Fazekas | ePVS vs WMH, for severe WMH comparing low (grade 1)/high (2-4) BG ePVS (*p*<.001) and low/high CSO ePVS (*p*<.001). | Positive |
| Yamada et al. (2019) | Healthy (N=296) |  | 66 **±** 6 | 1.5T/3T T2 visual count grade | 1.5T/3T T2 FLAIR Fazekas | CSO ePVS vs WMH, for predicting WMH from ePVS grades 1+ compared to 0 (*p*=0.009).  CSO ePVS vs WMH, for predicting ePVS from WMH grade 2-3 compared to 0 (*p*<.001). BG ePVS vs WMH, for predicting WMH for ePVS grades 1+ compared to 0 (*p*<.001). BG ePVS vs WMH, for predicting ePVS from WMH grease 1+ compared to 0 (*p*=.014-.001) | Negative  Negative  Positive  Positive |
| Yao et al. (2014 Nov) | CADASIL (N=344) |  | 51 **±** 12 | 1.5T T2/T2 FLAIR visual count grade | 1.5T T2 FLAIR volume | ePVS vs WMH, for temporal (OR=3.51, *p*<.001), subinsular (OR=2.42, *p*<.001) ePVS when comparing low (1-2 grade) and high (3-4 grade) ePVS. ePVS vs WMH, for BG (*p*=.88) and white matter *p*=.22() ePVS when comparing low/high ePVS. | Positive  Non-significant |
| Yao et al. (2014 Mar) | Healthy (N=1818) |  | 72 **±** 4 | 1.5T T2/T2 FLAIR visual count grade (Hippocampus) | 1.5T T2/T2 FLAIR volume | ePVS vs WMH, when comparing low (grade 0) to high (grade 2) ePVS (OR=1.29-1.35, *p*<.001). ePVS vs WMH, when comparing low to mid (grade 1) ePVS (OR=1.09-1.11, *p*=.10-.17). | Positive  Non-significant |
| Zeng et al. (2022) | Healthy (N=144) |  | 72 **±** 6 | T1 visual count grade | T2 FLAIR volume | BG ePVS vs WMH (β=0.200, *p*=.073). CSO ePVS vs WMH (β=0.257, *p*=.005). | Non-significant  Positive |
| Zhang K et al. (2022) | Healthy/CSVD (N=270) |  | 62 **±** 10 | T2 visual count grade (BG/CSO/Hippocampus) | T2 FLAIR Fazekas | BG ePVS vs WMH, across ePVS grade (*p*<.001). CSO/Hippocampal ePVS vs WMH, across ePVS grade (*p*=.060-.684). | Positive  Non-significant |
| Zhu et al. (2010) | Healthy (N=1818) |  | 73 **±** 4 | 1.5T T1/T2 visual count grade | 1.5T T2 volume | BG ePVS vs WMH, across ePVS grade (*p*<.0001). White matter ePVS vs WMH, across ePVS grade for deep WMH (*p*<.0001), but not for total (*p*=.02) or periventricular (*p*=.18) WMH. | Positive  Positive  Non-significant |
| ***BBB Permeability x WMH BBBP WMH Association Summary*** | | | | | | | |
| Bakhtiari et al. (2023) | Healthy (N=114) |  | 67 **±** 1 | Gadovist DCE Ki | T2 Fazekas | BBBP vs periventricular WMH, in the thalamus (p=.024). | Positive |
| Chen YC. et al. (2022) | T2DM (*n*=25) Non-T2DM (*n*=12) |  | 69 **±** 10 56 ± 19 | Gd-DOTA DCE ktrans | T2 FLAIR Fazekas | T2DM: BBBP vs WMH.  Non-T2DM: None BBBP vs WMH, unless ePVS ≥6 and WMH ≥1 (*p*=.024-.047) in caudate head or frontal white matter. | Non-significant  Positive |
| Dewey et al. (2021) | Healthy (N=21) |  | 54 **±** 4 | Gadoteridol DCE ktrans | T2 FLAIR location | BBBP vs WMH, when comparing WMH to NAWM (*p<.001)* | Unspecified |
| Dobrynina et al. (2022) | CSVD (n=53) Healthy (n=17) |  | 60 **±** 7  57 **±** 7 | gadodiamide DCE ktrans, Vp and AUC | T2 FLAIR Fazekas and location | BBBP vs WMH, when comparing Vp WMH and AUC WMH across Fazekas grades.  Ktrans was not different across Fazekas, and no differences in measures in NAWM. | Unspecified  Non-significant |
| Ford et al. (2022) | Healthy (N=20) |  | Range 25-65+ | diffusion weighted ASL kw | T2 FLAIR volume and location | BBBP vs WMH, in WMH (*r*=-0.51, *p*=.02). BBBP vs WMH, in NAWM (*r*=-0.44, *p*=.05). | Negative  Non-significant |
| Freeze et al. (2020) | Healthy (n=32) MCI (n=34) Dementia (n=14) |  | 72 **±** 7  69 **±** 6  72 **±** 7 | Gadavist DCE influx rate (Ki) and leakage volume (VL) | T2 FLAIR volume | Ki vs WMH (β=0.21-0.25, *p<*.039) in WMH, NAWM, deep grey, cortex, total white and total grey matter.  VL vs WMH (β=0.22-0.27, *p<*.03) in WMH, NAWM, deep grey, cortex, total white and total grey matter. BBBP vs WMH (β=0.11-0.12, *p>*.05), in the hippocampus. | Positive  Positive  Non-significant |
| Fujima et al. (2020) | Leukoaraiosis (n=41) Healthy (n=5) | Yes | 68 (range 50-76)  63 (range 58-68) | diffusion weighted ASL kw and kw-variance | T2 FLAIR category (non-lesion/ moderate/severe) | kw vs WMH (*p*=.42), when comparing lesion progressing to non-lesion progressing groups. Positive kw-variance vs WMH (*p*<.001) | Non-significant  Positive |
| Hillmer et al. (2022) | VCID/AD/ Leukoaraiosis (N=136) |  | 68 (Q1=62, Q3=75) | Gd-DTPA DCE ktrans | T1/T2 FLAIR volume | BBBP vs WMH (R^2^=0.172, *p*<.001) | Positive |
| Huisa et al. (2015) | CSVD (n=22) Healthy (n=12) | Yes | 67 **±** 10  61 **±** 10 | 1.5T/3T Magnevist DCE value threshold | 1.5T/3T FLAIR Fazekas and location | CSVD: BBBP vs WMH (*p*=.22), 51% of permeability voxels 4mm around WMH. Healthy: BBBP vs WMH, baseline BBBP preceded 11% of new WMH (*significance not reported*). | Non-significant  Unspecified |
| Kerkhofs et al. (2021) | Clinically Manifested CSVD (N=43) | Yes | 68 **±** 12 | gadobutrol DCE Ki and VL (volume leakage) | T2 FLAIR location | BBBP vs WMH, for both Ki (*p*=.001) and VL (*p*=.023) compared to NAWM.  BBBP vs WMH, comparing longitudinal Ki or VL with NAWM or WMH (β=-0.071-0.237, *p*=.130-.650). | Positive  Non-significant |
| Li et al. (2018) | Healthy (*N*=99) |  | 70 **±** 9 | gadolinium DCE ktrans | T2 FLAIR Fazekas (low/high), volume and location | BBBP vs WMH, for volume and Fazekas (R^2^=0.108-0.243, *p*<.001) in all areas of ktrans (NAWM, WMH, cortical grey, deep grey). | Unspecified |
| Li et al. (2017) | Healthy (N=102) |  | 70 **±** 9 | gadolinium DCE ktrans, AUC and Vp (plasma volume) | T2 FLAIR Fazekas | BBBP vs WMH, comparing ktrans/AUC across groups of low, medium and high WMH score in NAWM, WMH, cortical grey and deep grey matter (r=0.302-0.565, all *p*<.01). BBBP vs WMH, when comparing Vp across groups in NAWM, WMH, cortical grey and deep grey matter (*r*=-0.401- -0.265, all *p*<.01*)* | Positive  Negative |
| Manning et al. (2021; grey literature) | CSVD (N=80) |  | 69 | DCE PS | Not reported | BBBP vs WMH (*significance not reported*). | Non-significant |
| Shao et al. (2020) | Healthy/MCI (N=16) |  | 68 **±** 3 | Gd-DOTA DCE ktrans and DP-pCASL water exchange (kw) | T2 FLAIR location | BBBP vs WMH, for ktrans (*p*=.07) or kw (*p*=.09) comparing NAWM and WMH. | Non-significant |
| Shao et al. (2019) | Healthy/MCI (N=19) |  | 69 **±** 8 | DP-pCASL water exchange (kw) | T2 FLAIR volume and Fazekas | kw vs WMH, for Fazekas (β=10.61, *p*=.04). kw vs WMH, for WMH volume (β=1.68, *p*=.20). | Positive  Non-significant |
| Song et al. (2011) | Multiple System Atrophy (n=16) Healthy (n=13) |  | 59 **±** 8  61 **±** 10 | Gadovist DCE ktrans | T2 FLAIR volume | ktrans vs WMH, for ktrans in periventricular white matter (*r*=0.58, *p*=.019). | Unspecified |
| Starr et al. (2003) | Type 2 Diabetes (n=10) Healthy (n=10) |  | 68  68 | 1.9T Gd-DTPA DCE contrast enhancement | 1.9T T2 FLAIR Fazekas | BBBP vs WMH, for total WMH (*p*=.009), PVWMH (*p*=.023) and diffuse WMH (*p*=.005). | Positive |
| Zhang CE et al. (2019) | CSVD (n=77) Healthy (n=39) |  | 70 **±** 11  69 **±** 12 | DCE Gadobutrol Ki and VL (volume leakage) | T2 FLAIR volume | CSVD: Ki vs WMH (β=-0.267, *p*=.02). CSVD: VL vs WMH (β=0.268, *p*=.02). Healthy: Ki or VL vs WMH (β=0.159-0.184, *p*=.26-.33). | Negative Positive  Non-significant |
| ***Cerebral Perfusion x WMH Perfusion WMH Association Summary*** | | | | | | | |
| Abi Zeid Daou et al. (2018) | Depression (*n*=23) Healthy (*n*=20) |  | 68 **±** 7  68 **±** 6 | pCASL CBF and CVR to CO_2_ Challenge | T2 & T2 FLAIR volume | CBF vs WMH, for rostral anterior cingulate cortex (F_(1,38)_=7.64, *p*=.009) not other regions (*p>*.05). CVR vs WMH (*significance not reported*). | Negative  Non-significant |
| Anderson et al. (2014) | Healthy/MCI (N=20) |  | 71 **±** 6 | 7T gadoteridol DCE blood volume (Vb) | 7T T2 & T2 FLAIR Fazekas | Vb vs WMH, comparing to across NAWM, PWMH and DWMH (F_(2,14)_=6.33, *p*=.005), with Vb decreased in WMH. | Negative |
| Bahrani et al. (2017) | Healthy (N=26) |  | 78 **±** 7 | PASL CBF | T2 FLAIR segmentation and volume | CBF vs WMH, in both DWMH (*t*=5.7, *p*<.0001) and PWMH (*t*=11.0, *p*<.0001) compared to NAWM. Regional difference in correlations between WMH and CBF. | Negative |
| Bangen et al. (2021) | Healthy/MCI (N=147) | Yes | 72 **±** 7 | PASL CBF | T1/T2 FLAIR volume | CBF vs WMH, cross-sectional (*p*=.126-.425, *r*=0.07-0.12). CBF vs WMH, baseline entorhinal CBF predicting increase of WMH (*t*(96.87)=-2.21, *p*=.029, *r*=0.22), but not hippocampal CBF (*p*=.649, *r*=0.05). | Non-significant  Negative  Non-significant |
| Bastos-Leite et al. (2008) | Healthy/AD (N=21) |  | 76 **±** 5 | 1.5T PASL CBF | 1.5T T2 FLAIR Fazekas | CBF vs WMH, for global, subcortical and cortical CBF across low (grade 1-2) and high (grade 3) WMH (*p*<.05). | Negative |
| Bauer et al. (2021) | Healthy (N=80) |  | 70 **±** 6 | pCASL CBF | T2 FLAIR volume | CBF vs WMH (*F*=4.339, *p*=.041). | Negative |
| Benedictus et al. (2014) | AD (n=129) Healthy (n=61) |  | 66 **±** 7 64 **±** 5 | pCASL CBF | T1/T2 FLAIR volume | AD: CBF vs WMH (β=-0.24, *p*<.05). Healthy: CBF vs WMH (β=-0.21, *p*>.05). | Negative  Non-significant |
| Brickman et al. (2009) | Healthy (N=17) |  | 65 **±** 3 | 1.5T CASL CBF | 1.5T T2 FLAIR segmentation | CBF vs WMH, comparing across NAWM and WMH (*p*<.001). Lower CBF is predictive of higher WMH frequency (F_(14,21787)_=174.439, *p*<.001). | Negative |
| Cai et al. (2022) | Healthy (N=152) |  | 63 **±** 8 | PASL CBF | T2 FLAIR volumes | White matter CBF vs WMH (r=-0.163- -0.231, p=.004-.045)  Grey matter CBF vs WMH (*r*=-.073- -.101, *p*=.218-.375) | Negative  Non-significant |
| Callen et al. (2020) | HIV + CVD Risk (n=10) CVD Risk Only (n=7) |  | 57 **±** 11  56 **±** 5 | phase-contrast/ASL CBF | T2 FLAIR modified Fazekas | CBF vs WMH (*B*=40.8, *p*<.001). | Unspecified |
| Chai et al. (2018) | Tetralogy of Fallot (N=46) |  | 37 **±** 14 | pCASL CBF | T2 FLAIR segmentation | CBF vs WMH, comparing NAWM to WMH (*p*=.07). | Non-significant |
| Crane et al. (2015) | Healthy/MCI with WMH |  | 73 **±** 9 | pCASL CBF | T2 FLAIR volume | CBF vs WMH, in several regions (*rho*=-0.61- -0.42, *p*=.002-.040). | Negative |
| Dalby et al. (2019) | Depression (n=21) Healthy (n=21) | Yes | 57 **±** 5  58 **±** 7 | Gadovist DSC CBF, CBV and MTT | T2 FLAIR location | CBF vs WMH, comparing NAWM to WMH (*t*=-4.97, *p*<.0001).  CBV vs WMH (*t*=-14.99, *p*<.0001). MTT vs WMH, comparing NAWM to WMH (t=2.36, *p*=.0238). | Negative  Negative Positive |
| Dolui et al. (2019) | Healthy Adult (n=436) Healthy Elderly (n=61) |  | 50 **±** 4  73 **±** 7 | pCASL CBF | T2 FLAIR location | Healthy Adult: CBF vs WMH, comparing WMH to NAWM (partial η2=0.59, p<.0001) Healthy Elderly: CBF vs WMH, comparing WMH to NAWM (partial η2=0.73, p<.0001) | Negative  Negative |
| Eskildsen et al. (2017) | Possible MCI/AD (n=18) |  | 71 **±** 7 | 1.5T Gadovist DSC relative transit time heterogeneity (RTH), capillary transit time heterogeneity (CTH), MTT | 1.5T T2 FLAIR | RTH vs WMH in temporal, parietal and frontal lobes (p<.05)  CTH vs WMH, in anterior cingulate cortex (p<.05)  MTT vs WMH, from in cortex (p<.05) | Positive  Positive  Negative |
| Ford et al. (2022) | Healthy (N=20) |  | Range 25-65+ | diffusion weighted ASL CBF | T2 FLAIR volume | CBF vs WMH, in WMH (*r*=-0.49, *p*=.03). CBF vs WMH, in NAWM (*r*=-0.40, *p*=.08). | Negative  Non-significant |
| Fujima et al. (2020) | Leukoaraiosis (n=41) Healthy (n=5) | Yes | 68 (range: 50-76)  63 (range: 58-68) | diffusion weighted ASL CBF | T2 FLAIR category (non-lesion/ moderate/severe) | CBF vs WMH (*p*,.001), when comparing lesion progressing to non-lesion progressing groups. | Negative |
| Gyanwali et al. (2022 Oct) | Mixed Sample of CVD/Clinical Diagnoses (N=333) |  | median=73, IQR=10 | pCASL CBF and spatial coefficient of CBF variation (sCoV) | T2 FLAIR volume | Full sample: sCoV vs WMH (β=0.24, *p*<.001). Full sample: CBF vs WMH (β=-0.07- -0.10, *p*=.057-.164). Normal: sCoV vs WMH (β=0.20, *p*=.049).  Normal: CBF vs WMH (β=-0.12-0.02, *p*=.365-.863).  MCI: sCoV vs WMH (β=0.19, *p*=.002) MCI: CBF vs WMH (β=-0.03- -0.13, *p*=.112-.644). Dementia: CBF vs WMH (p>.05).  Dementia: sCoV vs WMH (p>.05). | Positive  Non-significant  Positive  Non-significant  Positive  Non-significant Non-significant  Non-significant |
| Gyanwali et al. (2022 Dec) | Mixed Healthy/MCI/AD (N=368) | Yes | median=73, IQR=11 | pCASL CBF and spatial coefficient of CBF variation (sCoV) | T2 FLAIR volume | sCoV vs WMH, sCoV associated with longitudinal WMH progression (Wald=3.88, *p*=.049). | Positive |
| Han & Lin et al. (2022) | Healthy (N=127) | Yes | 69 **±** 7 | phase-contrast CBF | T2 FLAIR volume | CBF vs WMH, when comparing change from baseline CBF and change from baseline WMH. (*r*=-0.25, *p*=.011). | Negative |
| Han & Ning et al. (2022) | Healthy (N=229) | Yes | 57 **±** 13 | pCASL CBF | T2 FLAIR volume | CBF vs WMH, comparing to NAWM (*p*<.001).  CBF vs WMH, within WMH lesions (B=-0.017, *p*=.016). CBF vs WMH, WMH progression vs CBF change  (B=-1.01, *p*=.015) | Negative  Negative Negative |
| Huang et al. (2022) | Healthy (n=65) With WMH (n=65) |  | 58 (range 55-65)  63 (range 56-68) | pCASL CBF | T2 FLAIR Fazekas | CBF vs WMH, in left orbital medial frontal gyrus, left middle temporal gyrus and right thalamus when comparing groups (p<.05). CBF vs WMH, in left triangular inferior front gyrus when comparing groups (p<.05). | Negative  Positive |
| Huang et al. (2021) | Mild WMH (n=32) Moderate WMH (n=24) Severe WMH (n=30) |  | 64 **±** 5  64 **±** 7  66 **±** 5 | pCASL CBF | T2 FLAIR Fazekas | CBF vs WMH, when comparing groups in many regions (*p*<.01). | Negative |
| Jann et al. (2021) | Vascular Cognitive Impairment and Dementia (N=45) |  | 69 **±** 7 | pCASL CBF (in white matter and leptopmeningeal middle cerebral artery (leptoMCA)) | T2 FLAIR Fazekas | CBF vs WMH, for white matter CBF vs WMH volume (β=-0.37, *p*=.01) and Fazekas score (*p*=.03).  CBF vs WMH volume, for leptoMCA CBF vs WMH volume (β=0.37, *p*=.01) and Fazekas (*p*=.02). | Negative  Positive |
| Kang et al. (2022) | Healthy (n=42) |  | 66 (range 57-73) | pCASL CBF | T2 FLAIR volume and location | CBF vs WMH, for white matter (ρ=-0.126, *p*=.432) and watershed CBF (ρ=-0.202, *p*=.205). CBF vs WMH, CBF decreases approaching WMH (from inside, to 4mm to 8mm; *p*<.0001). | Non-significant   Negative |
| Kim et al. (2020) | MCI (n=17) Healthy (n=21) |  | 68 ± 6  68 **±** 6 | pCASL CBF | T1 volume | MCI: CBF vs WMH in many regions (*p*<.05). Healthy: CBF vs WMH (*p*>.05). | Negative  Non-significant |
| Kim et al. (2022) | Healthy (N=254) |  | 50 **±** 4 | pCASL CBF | T2 FLAIR volume | CBF vs WMH, comparing low to high (7-10^th^ decile) WMH volume (*p*=.44). | Non-significant |
| Knoops et al. (2009) | Healthy (N=392) |  | 62 **±** 9 | phase-contrast parenchymal CBF | T2 FLAIR volume | CBF vs WMH, for interaction term (*p*=.84). | Non-significant |
| Liem et al. (2009) | NOTCH3 carriers (n=25) Healthy (n=13) |  | 42 **±** 10  37 **±** 8 | 1.5T phase contrast CBF and CVR to acetazolamide | 1.5T T2 FLAIR volume | CBF vs WMH, when comparing low to high total CBF to WMH change (*p*>.05). CVR vs WMH, when comparing low to high CVR to WMH change (*p*=.016). | Non-significant  Negative |
| Lu et al. (2022) | CSVD (n=121) Healthy (n=53) |  | 64 **±** 9  57 **±** 9 | pCASL CBF pattern category (heterogeneity through discriminative analysis) | T2 FLAIR Fazekas | CBF vs WMH, comparing difference of PVWMH between three pattern groups (χ2 = 6.316, *p*=.043). CBF vs WMH, when comparing difference of total or deep WMH (all *p*>.05) | Unspecified    Non-significant |
| Marstrand et al. (2002) | Healthy (N=21) |  | 86 (range 85-86) | 1.5T Gd-DTPA DSC CBF MTT and CBV, and CVR to acetazolamide | 1.5T T2 FLAIR location | CBF/MTT vs WMH, comparing NAWM to WMH (*p*=.004, *p*<.001).  Negative CVR vs WMH, comparing CBF change in NAWM to WMH (*p*=.026). CBV vs WMH, comparing NAWM to WMH. | Negative  Negative   Non-significant |
| Nasel et al. (2017) | Adult Low WMH (n=18) Elderly Low WMH (n=38) Elderly CSVD (n=50) |  | 42 **±** 9  68 **±** 9  76 **±** 10 | 1.5T DSC Gadoteridol/Gd-DOTA bolus spread velocity | 1.5T T2 volume | Perfusion vs WMH (R^2^=0.2696, *p*<.001). | Negative |
| Ni et al. (2020) | ICAS (N=41) |  | 57 **±** 10 | DSC Gd-DTPA time-to-peak (TTP) | T2 FLAIR volume | TTP vs WMH, for both inter-hemispheric (β=-0.613, *p*<.001) and ipsilateral deep WMH (β=-0.428, *p*=.005), and total WMH ipsilateral to ICAS site (β=-0.343, *p*=.014). TTP vs WMH, for PVWMH (*p*>.05). | Negative  Non-significant |
| Nylander et al. (2018) | Healthy (N=406) | Yes | 75 **±** 0 | 1.5T DSC gadolinium regional CBF | 1.5T T1/T2 modified Fazekas | CBF vs WMH, for both cross-sectional or change in WMH with white matter CBF (*significance not reported*). | Non-significant |
| Onkenhout et al. (2020) | CSVD (N=132) |  | 73 **±** 10 | phase-contrast CBF | T2 FLAIR volume and Fazekas | CBF vs WMH, for both Fazekas (*p*=.07) and volume (*p*=.20) | Non-significant |
| O’Sullivan et al. (2002) | Leukoaraiosis (n=21) Healthy (n=15) |  | 69 **±** 9  72 **±** 8 | 1.5T angiogram Gadolinium CBF | 1.5T T2 FLAIR location | Leukoaraiosis: CBF vs WMH, when comparing NAWM to WMH for mean periventricular CBF (*p*<.05) and mean CSO CBF (*p*<.05), but not for more region specific CBF (*p*>.05). | Negative  Non-significant |
| Pahlavian et al. (2021) | Healthy/MCI (N=50) |  | 69 **±** 7 | pCASL CBF and phase-contrast ICA flow, pulsatility and relativity index | T2 FLAIR Fazekas and volume | Pulsatility/Relativity vs WMH, for deep WMH score (β=0.17-0.05, *p*=.01) and total volume (β=8.32-32.3, *p*=.01). Pulsatility/Relativity vs WMH, for PVWMH (*p*>.05). Mean ICA flow/CBF vs WMH, for any WMH region (*p*>.05). | Positive  Non-significant  Non-significant |
| Phyu et al. (2018) | Fabry Disease (n=25) |  | 42 (range 26-70) | PASL CBF | T2 volume | CBF vs WMH (*r*=0.59, *p*=.006). | Unspecified |
| Promjunyakul et al. (2015) | Healthy (N=61) |  | 85 **±** 8 | PASL CBF | T2 FLAIR volume | CBF vs WMH, when comparing PVWMH or DWMH to NAWM (*p*<.001). CBF vs WMH, when comparing baseline CBF between new WMH voxels to persistent NAWM voxels (*p*=.006). | Negative  Negative |
| Promjunyakul et al. (2018) | Healthy (N=52) |  | 83 **±** 8 | PASL CBF | T2 FLAIR volume | CBF vs WMH, when comparing baseline CBF between new WMH voxels to persistent NAWM voxels (*p*<.001). CBF vs WMH, CBF associated with WMH growth (GEE estimate=-2.29, *p*=.022). | Negative  Negative |
| Rane et al. (2018) | Healthy/MCI (N=28) |  | 76 **±** 7 | pCASL CBF | T2 FLAIR location | CBF vs WMH, as voxels approach WMH (R^2^=0.98, cubic fit). | Negative |
| Rane et al. (2020) | Mixed Healthy/MCI (N=103) |  | 75 **±** 9 | pCASL CBF | T1/T2 FLAIR count | CBF vs WMH, for both PVWMH and DWMH (*significance not reported*). | Negative |
| Salomonsson et al. (2023) | Female SLE (N=64) |  | median=39  (range 18-52) | DSC CBF, CBV, MTT, leakage correction (K2) | T2 FLAIR location | Perfusion vs WMH (n=31), for CBF/CBV/MTT/K2 when comparing WMH to NAWM (*21.4-231.4% increase, p<.005)*. | Positive |
| Sam et al. (2016) | Leukoaraiosis (N=75) |  | 74 **±** 10 | gadolinium DSC CBF, MTT, TTP and regional CBV | T2 FLAIR location | Perfusion vs WMH, comparing NAWM to WMH for CBF (*t*=2.8, *p*=.01) and CBV (*t*=2.3, *p*=.03).  Perfusion vs WMH, comparing NAWM to WMH for MTT (*t*=1.5, *p*=.15) and TTP (*p*<.001). | Negative   Non-significant |
| Shi et al. (2020) | Minor stroke (N=56) |  | 68 **±** 8 | 1.5T phase-contrast CBF and carotid pulsatility index (PI) | T2 FLAIR volume and Fazekas | CBF vs WMH, comparing Fazekas grades (*p*=.250-.476) or volume. PI vs WMH, for WMH volume (β=0.841, *p*=.038) | Non-significant   Negative |
| Shi et al. (2017) | Healthy (N=69) |  | 71 **±** 4 | pCASL CBF | T2 FLAIR volume | CBF vs WMH, with left superior motor region CBF and right superior corona radiata WMH (*r*=0.42, *p*<.001), and right putamen region CBF and left anterior limb of internal capsule WMH (*r*=0.43, *p*<.001), | Positive |
| Staffaroni et al. (2019) | Healthy (N=161) |  | 70 **±** 8 | PASL CBF | T2 FLAIR count | CBF vs WMH (*b*=-0.02, *p*=.007). | Negative |
| Tarumi et al. (2014) | Healthy (N=24) |  | 66 **±** 5 | phase-contrast CBF | T2 FLAIR volume | CBF vs WMH (R^2^=0.03, *p*=0.42). | Non-significant |
| Tu et al. (2022) | Subcortical ischemic vascular disease (n=42) AD (n=50) |  | 73 **±** 9  77 **±** 5 | pCASL CBF | T2 FLAIR count | SIVD: CBF vs WMH, in various regions (*p*<.05). AD: CBF vs WMH, in left lentiform nucleus CBF and frontal/temporal WMH (*r*=.490, *p*=.021) in those with CDR=0.5. AD: CBF vs WMH, in left inferior frontal gyrus CBF and BG WMH (*r*=-0.382, *p*=.045) in those with CDR=1-2. | Negative  Positive  Negative |
| Uh et al. (2010) | Leukoaraiosis (n=10) |  | not reported | pCASL CBF | T2 FLAIR segmentation | CBF vs WMH, compared to NAWM (*p*<.001). | Negative |
| van Dalen et al. (2016) | Hypertension (N=181) |  | 77 **±** 2 | pCASL CBF | T2 FLAIR segmentation | CBF vs WMH, compared to NAWM (*p*<.001) and comparing low quartile to high quartile WMH (*p*=.007). | Negative |
| van der Veen et al. (2015) | Manifest arterial disease (N=575) | Yes | 57 **±** 10 | 1.5T phase-contrast CBF | 1.5T T2 FLAIR volume | CBF vs WMH, for CBF associated WMH progression (*B*=0.00, 95% CI = -0.05 to 0.05, *p*>.05).  CBF vs WMH, for WMH associated with CBF decline (*B*=-0.70, 95% CI = -1.40 to 0.00, *p*<.05). | Non-significant  Negative |
| van Es et al. (2010) | History or risk of vascular disease (N=464) |  | 75 **±** 3 | 1.5T phase-contrast tCBF (mL/min) and CBF (mL/100 mL/min) | 1.5T FLAIR volume | tCBF vs WMH (*r*=-.069, *p*=.148). CBF vs WMH (*r*=-.106, *p*=.044). | Non-significant  Negative |
| Wang R et al. (2022) | CADASIL (n=12) Healthy (n=6) |  | 52 **±** 16  46 **±** 11 | pCASL CBF | T2 FLAIR volume | CBF vs total WMH, for CBF in WMH (*r*=-.710, *p*=.001), NAWM (*r*=-.586, *p*=.011), grey matter (*r*=-.545, *p*=.019) and global (*r*=-.748, *p*<.001). | Negative |
| Zhang Q et al. (2017) | Migraine (n=60) Migraine w/Aura (n=56) Healthy (n=54) |  | 34 **±** 7  34 **±** 7  32 **±** 6 | pCASL CBF | T2 FLAIR count | Migraine: CBF vs WMH (*p*=.98). Migraine w/Aura: CBF vs WMH (*p*=.03). Healthy: CBF vs WMH (*p*=.74). | Non-significant  Negative  Non-significant |
| Zhang R et al. (2022) | CSVD (N=92) |  | 65 **±** 10 | PASL CBF (for PLD 1.5, PLD 2.0) | T2 FLAIR volume | CBF vs WMH (β=-0.409- -0.483, *p*<.001). | Negative |
| Zhang W et al. (2022) | SIVD (n=38) SIVD-MCI (n=36) |  | 62 **±** 5  63 **±** 6 | pCASL CBF | T2 FLAIR volume | SIVD: CBF vs WMH, for whole-brain CBF (*r*=-0.344, *p*=.043) but not for grey matter (*r*=-0.247, *p*=.153) or white matter (*r*=-0.182, *p*=.296) CBF. SIVD-MCI: CBF vs WMH, for whole (*r*=-0.357, *p*=.041) and grey matter (*r*=-0.337, *p*=.045) CBF, not for white matter (*r*=-0.270, *p*=.112). | Negative  Non-significant  Negative  Non-significant |

***Supplement Table 1****.* Included studies investigating the relationship between key MR markers and WMH, their study design, and a qualitative summary of their findings.
Marker associations are summarised as either ‘positive’, ‘negative’, ‘unspecified’ (analysis does not detail relationship direction) or ‘non-significant’ (did not meet statistical significance). Strength and statistical significance of relationship reported where available. Models adjusted for age and sex, or minimal models reported.

Age is reported from baseline statistics in longitudinal studies.

AD = Alzheimer’s disease; ATT = arterial transit time; AUC = area under the curve of increase in contrast transit time; BBB = blood brain barrier; BBBP = blood brain barrier permeability;
BG = basal ganglia; CAA = cerebral amyloid angiopathy; CADASIL = cerebral autosomal dominant arteriopathy with subcortical infarcts and leukoencephalopathy; CASL = continuous arterial spin labelling; CBF = cerebral blood flow; CDR = clinical dementia rating scale; CMB = cerebral microbleed; CSO = centrum semiovale; CSVD = cerebral small vessel disease;
DP-pCASL = diffusion prepared pseudo continuous arterial spin labelling; DSC = dynamic susceptibility contrast; ePVS = enlarged perivascular space; FLAIR = fluid attenuated inversion recovery; Gd-DOTA = gadoterate meglumine; Gd-DTPA = gadolinium diethyltriamine pentaacetic acid; ICAS = intracranial atherosclerotic stenosis; ICH = intracerebral hemorrhage;
ktrans = volume transfer constant of contrast into brain tissue (leakage rate); kw = water exchange rate; MCI = mild cognitive impairment; MTT = mean transit time; PASL = pulsed arterial spin labelling; pCASL = pseudo-continuous arterial spin labelling; PLD= post-labelling delay; SIVD = subcortical ischemic vascular disease; TIA = transient ischemic attack; Vp = fractional blood plasma volume of contrast; WMH = white matter hyperintensity.

*B* = unstandardised regression coefficient; β = standardised regression coefficient; *F* = F ratio; *p* = significance value; partial η2 = partial eta squared; *OR* = odds ratio; r = Pearson correlation coefficient; *R^2^* = coefficient of determination; *rho* = Spearman’s rank correlation coefficient; *t* = independent t-test value; χ2 = chi-squared.

* “Healthy” refers to samples that are comprised of participants that do not have a specific disease or are without significant neurological disease, cognitive impairment or insult.
^ MRI is 3T unless otherwise specified.
# ePVS is quantified in the BG and CSO unless otherwise specified. ePVS “grade”: 0= no visual ePVS, 1= <10, 2= 10–20, 3= 20–40, 4= >40. Fazekas grade range 0-4. Scheltens grade range 0-6.

**References:**

Abi Zeid Daou, M., Boyd, B. D., Donahue, M. J., Albert, K., & Taylor, W. D. (2018, Feb). Anterior-posterior gradient differences in lobar and cingulate cortex cerebral blood flow in late-life depression. *J Psychiatr Res, 97*, 1-7. https://doi.org/10.1016/j.jpsychires.2017.11.005

Anderson, V. C., Obayashi, J. T., Kaye, J. A., Quinn, J. F., Berryhill, P., Riccelli, L. P., Peterson, D., & Rooney, W. D. (2014). Longitudinal relaxographic imaging of white matter hyperintensities in the elderly. *Fluids Barriers CNS, 11*, 24. https://doi.org/10.1186/2045-8118-11-24

Arba, F., Quinn, T. J., Hankey, G. J., Lees, K. R., Wardlaw, J. M., Ali, M., Inzitari, D., & Collaboration, V. (2018, Jan). Enlarged perivascular spaces and cognitive impairment after stroke and transient ischemic attack. *Int J Stroke, 13*(1), 47-56. https://doi.org/10.1177/1747493016666091

Aribisala, B. S., Wiseman, S., Morris, Z., Valdes-Hernandez, M. C., Royle, N. A., Maniega, S. M., Gow, A. J., Corley, J., Bastin, M. E., Starr, J., Deary, I. J., & Wardlaw, J. M. (2014, Feb). Circulating inflammatory markers are associated with magnetic resonance imaging-visible perivascular spaces but not directly with white matter hyperintensities. *Stroke, 45*(2), 605-607. https://doi.org/10.1161/STROKEAHA.113.004059

Bahrani, A. A., Powell, D. K., Yu, G., Johnson, E. S., Jicha, G. A., & Smith, C. D. (2017, Apr). White Matter Hyperintensity Associations with Cerebral Blood Flow in Elderly Subjects Stratified by Cerebrovascular Risk. *J Stroke Cerebrovasc Dis, 26*(4), 779-786. https://doi.org/10.1016/j.jstrokecerebrovasdis.2016.10.017

Bakhtiari, A., Vestergaard, M. B., Benedek, K., Fagerlund, B., Mortensen, E. L., Osler, M., Lauritzen, M., Larsson, H. B. W., & Lindberg, U. (2023, Apr). Changes in hippocampal volume during a preceding 10-year period do not correlate with cognitive performance and hippocampal blood‒brain barrier permeability in cognitively normal late-middle-aged men. *Geroscience, 45*(2), 1161-1175. https://doi.org/10.1007/s11357-022-00712-2

Ballerini, L., Booth, T., Valdes Hernandez, M. D. C., Wiseman, S., Lovreglio, R., Munoz Maniega, S., Morris, Z., Pattie, A., Corley, J., Gow, A., Bastin, M. E., Deary, I. J., & Wardlaw, J. (2020). Computational quantification of brain perivascular space morphologies: Associations with vascular risk factors and white matter hyperintensities. A study in the Lothian Birth Cohort 1936. *Neuroimage Clin, 25*, 102120. https://doi.org/10.1016/j.nicl.2019.102120

Bangen, K. J., Thomas, K. R., Sanchez, D. L., Edmonds, E. C., Weigand, A. J., Delano-Wood, L., Bondi, M. W., & Alzheimer's Disease Neuroimaging, I. (2021). Entorhinal Perfusion Predicts Future Memory Decline, Neurodegeneration, and White Matter Hyperintensity Progression in Older Adults. *J Alzheimers Dis, 81*(4), 1711-1725. https://doi.org/10.3233/JAD-201474

Barnes, A., Ballerini, L., Valdes Hernandez, M. D. C., Chappell, F. M., Munoz Maniega, S., Meijboom, R., Backhouse, E. V., Stringer, M. S., Duarte Coello, R., Brown, R., Bastin, M. E., Cox, S. R., Deary, I. J., & Wardlaw, J. M. (2022). Topological relationships between perivascular spaces and progression of white matter hyperintensities: A pilot study in a sample of the Lothian Birth Cohort 1936. *Front Neurol, 13*, 889884. https://doi.org/10.3389/fneur.2022.889884

Bastos-Leite, A. J., Kuijer, J. P., Rombouts, S. A., Sanz-Arigita, E., van Straaten, E. C., Gouw, A. A., van der Flier, W. M., Scheltens, P., & Barkhof, F. (2008, Aug). Cerebral blood flow by using pulsed arterial spin-labeling in elderly subjects with white matter hyperintensities. *AJNR Am J Neuroradiol, 29*(7), 1296-1301. https://doi.org/10.3174/ajnr.A1091

Bauer, C. E., Zachariou, V., Seago, E., & Gold, B. T. (2021). White Matter Hyperintensity Volume and Location: Associations With WM Microstructure, Brain Iron, and Cerebral Perfusion. *Front Aging Neurosci, 13*, 617947. https://doi.org/10.3389/fnagi.2021.617947

Benedictus, M. R., Binnewijzend, M. A. A., Kuijer, J. P. A., Steenwijk, M. D., Versteeg, A., Vrenken, H., Scheltens, P., Barkhof, F., van der Flier, W. M., & Prins, N. D. (2014, Dec). Brain volume and white matter hyperintensities as determinants of cerebral blood flow in Alzheimer's disease. *Neurobiol Aging, 35*(12), 2665-2670. https://doi.org/10.1016/j.neurobiolaging.2014.06.001

Bouvy, W. H., Zwanenburg, J. J. M., Reinink, R., Wisse, L. E. M., Luijten, P. R., Kappelle, L. J., Geerlings, M. I., Biessels, G. J., & Utrecht Vascular Cognitive Impairment Study, g. (2016, Oct). Perivascular spaces on 7 Tesla brain MRI are related to markers of small vessel disease but not to age or cardiovascular risk factors. *J Cereb Blood Flow Metab, 36*(10), 1708-1717. https://doi.org/10.1177/0271678X16648970

Brickman, A. M., Zahra, A., Muraskin, J., Steffener, J., Holland, C. M., Habeck, C., Borogovac, A., Ramos, M. A., Brown, T. R., Asllani, I., & Stern, Y. (2009, May 15). Reduction in cerebral blood flow in areas appearing as white matter hyperintensities on magnetic resonance imaging. *Psychiatry Res, 172*(2), 117-120. https://doi.org/10.1016/j.pscychresns.2008.11.006

Cai, J., Sun, J., Chen, H., Chen, Y., Zhou, Y., Lou, M., & Yu, R. (2022). Different mechanisms in periventricular and deep white matter hyperintensities in old subjects. *Front Aging Neurosci, 14*, 940538. https://doi.org/10.3389/fnagi.2022.940538

Callen, A. L., Dupont, S. M., Pyne, J., Talbott, J., Tien, P., Calabrese, E., Saloner, D., Chow, F. C., & Narvid, J. (2020, Oct). The regional pattern of abnormal cerebrovascular reactivity in HIV-infected, virally suppressed women. *J Neurovirol, 26*(5), 734-742. https://doi.org/10.1007/s13365-020-00859-8

Chai, Y., Chen, J., Galarza, C., Sluman, M. A., Xu, B., Vu, C. Q., Richard, E., Mulder, B., Tamrazi, B., Lepore, N., Mutsaerts, H., & Wood, J. C. (2018, Apr). Cerebral Blood Flow and Predictors of White Matter Lesions in Adults with Tetralogy of Fallot. *Proc IEEE Int Symp Biomed Imaging, 2018*, 1309-1312. https://doi.org/10.1109/ISBI.2018.8363812

Charidimou, A., Boulouis, G., Haley, K., Auriel, E., van Etten, E. S., Fotiadis, P., Reijmer, Y., Ayres, A., Vashkevich, A., Dipucchio, Z. Y., Schwab, K. M., Martinez-Ramirez, S., Rosand, J., Viswanathan, A., Greenberg, S. M., & Gurol, M. E. (2016, Feb 9). White matter hyperintensity patterns in cerebral amyloid angiopathy and hypertensive arteriopathy. *Neurology, 86*(6), 505-511. https://doi.org/10.1212/WNL.0000000000002362

Charidimou, A., Boulouis, G., Pasi, M., Auriel, E., van Etten, E. S., Haley, K., Ayres, A., Schwab, K. M., Martinez-Ramirez, S., Goldstein, J. N., Rosand, J., Viswanathan, A., Greenberg, S. M., & Gurol, M. E. (2017, Mar 21). MRI-visible perivascular spaces in cerebral amyloid angiopathy and hypertensive arteriopathy. *Neurology, 88*(12), 1157-1164. https://doi.org/10.1212/WNL.0000000000003746

Crane, D. E., Black, S. E., Ganda, A., Mikulis, D. J., Nestor, S. M., Donahue, M. J., & MacIntosh, B. J. (2015). Gray matter blood flow and volume are reduced in association with white matter hyperintensity lesion burden: a cross-sectional MRI study. *Front Aging Neurosci, 7*, 131. https://doi.org/10.3389/fnagi.2015.00131

Dalby, R. B., Eskildsen, S. F., Videbech, P., Frandsen, J., Mouridsen, K., Sorensen, L., Jeppesen, P., Bek, T., Rosenberg, R., & Ostergaard, L. (2019). Oxygenation differs among white matter hyperintensities, intersected fiber tracts and unaffected white matter. *Brain Commun, 1*(1), fcz033. https://doi.org/10.1093/braincomms/fcz033

De Guio, F., Vignaud, A., Chabriat, H., & Jouvent, E. (2018, Sep). Different types of white matter hyperintensities in CADASIL: Insights from 7-Tesla MRI. *J Cereb Blood Flow Metab, 38*(9), 1654-1663. https://doi.org/10.1177/0271678X17690164

Del Brutto, O. H., Mera, R. M., Costa, A. F., Rumbea, D. A., Recalde, B. Y., & Del Brutto, V. J. (2022, Nov). Patterns of progression of cerebral small vessel disease markers in older adults of Amerindian ancestry: a population-based, longitudinal prospective cohort study. *Aging Clin Exp Res, 34*(11), 2751-2759. https://doi.org/10.1007/s40520-022-02223-8

Dewey, B. E., Xu, X., Knutsson, L., Jog, A., Prince, J. L., Barker, P. B., van Zijl, P. C. M., Leigh, R., & Nyquist, P. (2021, Aug). MTT and Blood-Brain Barrier Disruption within Asymptomatic Vascular WM Lesions. *AJNR Am J Neuroradiol, 42*(8), 1396-1402. https://doi.org/10.3174/ajnr.A7165

Ding, J., Sigurethsson, S., Jonsson, P. V., Eiriksdottir, G., Charidimou, A., Lopez, O. L., van Buchem, M. A., Guethnason, V., & Launer, L. J. (2017, Sep 1). Large Perivascular Spaces Visible on Magnetic Resonance Imaging, Cerebral Small Vessel Disease Progression, and Risk of Dementia: The Age, Gene/Environment Susceptibility-Reykjavik Study. *JAMA Neurol, 74*(9), 1105-1112. https://doi.org/10.1001/jamaneurol.2017.1397

Dobrynina, L. A., Shamtieva, K. V., Kremneva, E. I., Zabitova, M. R., Akhmetzyanov, B. M., Gnedovskaya, E. V., & Krotenkova, M. V. (2022, May 11). Daily blood pressure profile and blood-brain barrier permeability in patients with cerebral small vessel disease. *Sci Rep, 12*(1), 7723. https://doi.org/10.1038/s41598-022-11172-1

Dolui, S., Tisdall, D., Vidorreta, M., Jacobs, D. R., Jr., Nasrallah, I. M., Bryan, R. N., Wolk, D. A., & Detre, J. A. (2019). Characterizing a perfusion-based periventricular small vessel region of interest. *Neuroimage Clin, 23*, 101897. https://doi.org/10.1016/j.nicl.2019.101897

Duperron, M. G., Tzourio, C., Sargurupremraj, M., Mazoyer, B., Soumare, A., Schilling, S., Amouyel, P., Chauhan, G., Zhu, Y. C., & Debette, S. (2018, Feb). Burden of Dilated Perivascular Spaces, an Emerging Marker of Cerebral Small Vessel Disease, Is Highly Heritable. *Stroke, 49*(2), 282-287. https://doi.org/10.1161/STROKEAHA.117.019309

Eskildsen, S. F., Gyldensted, L., Nagenthiraja, K., Nielsen, R. B., Hansen, M. B., Dalby, R. B., Frandsen, J., Rodell, A., Gyldensted, C., Jespersen, S. N., Lund, T. E., Mouridsen, K., Braendgaard, H., & Ostergaard, L. (2017, Feb). Increased cortical capillary transit time heterogeneity in Alzheimer's disease: a DSC-MRI perfusion study. *Neurobiol Aging, 50*, 107-118. https://doi.org/10.1016/j.neurobiolaging.2016.11.004

Esnr 2022. (2022, Sep). *Neuroradiology, 64*(Suppl 1), 1-165. https://doi.org/10.1007/s00234-022-03012-w

ESOC 2022 Abstract Book. (2022). *European Stroke Journal, 7*(1_suppl), 3-545. https://doi.org/10.1177/23969873221087559

Evans, T. E., Knol, M. J., Schwingenschuh, P., Wittfeld, K., Hilal, S., Ikram, M. A., Dubost, F., van Wijnen, K. M. H., Katschnig, P., Yilmaz, P., de Bruijne, M., Habes, M., Chen, C., Langer, S., Volzke, H., Ikram, M. K., Grabe, H. J., Schmidt, R., Adams, H. H. H., & Vernooij, M. W. (2023, Jan 10). Determinants of Perivascular Spaces in the General Population: A Pooled Cohort Analysis of Individual Participant Data. *Neurology, 100*(2), e107-e122. https://doi.org/10.1212/WNL.0000000000201349

Ford, J. N., Zhang, Q., Sweeney, E. M., Merkler, A. E., de Leon, M. J., Gupta, A., Nguyen, T. D., & Ivanidze, J. (2022). Quantitative Water Permeability Mapping of Blood-Brain-Barrier Dysfunction in Aging. *Front Aging Neurosci, 14*, 867452. https://doi.org/10.3389/fnagi.2022.867452

Freeze, W. M., Jacobs, H. I. L., de Jong, J. J., Verheggen, I. C. M., Gronenschild, E., Palm, W. M., Hoff, E. I., Wardlaw, J. M., Jansen, J. F. A., Verhey, F. R., & Backes, W. H. (2020, Jan). White matter hyperintensities mediate the association between blood-brain barrier leakage and information processing speed. *Neurobiol Aging, 85*, 113-122. https://doi.org/10.1016/j.neurobiolaging.2019.09.017

Fujima, N., Kameda, H., Shimizu, Y., Harada, T., Tha, K. K., Yoneyama, M., & Kudo, K. (2020, Jun). Utility of a diffusion-weighted arterial spin labeling (DW-ASL) technique for evaluating the progression of brain white matter lesions. *Magn Reson Imaging, 69*, 81-87. https://doi.org/10.1016/j.mri.2020.03.005

Gertje, E. C., van Westen, D., Panizo, C., Mattsson-Carlgren, N., & Hansson, O. (2021, Jan 12). Association of Enlarged Perivascular Spaces and Measures of Small Vessel and Alzheimer Disease. *Neurology, 96*(2), e193-e202. https://doi.org/10.1212/WNL.0000000000011046

Gyanwali, B., Mutsaerts, H. J., Tan, C. S., Kaweilh, O. R., Petr, J., Chen, C., & Hilal, S. (2022, Dec). Association of Arterial Spin Labeling Parameters With Cognitive Decline, Vascular Events, and Mortality in a Memory-Clinic Sample. *Am J Geriatr Psychiatry, 30*(12), 1298-1309. https://doi.org/10.1016/j.jagp.2022.06.007

Gyanwali, B., Tan, C. S., Petr, J., Escobosa, L. L. T., Vrooman, H., Chen, C., Mutsaerts, H. J., & Hilal, S. (2022, Oct). Arterial Spin-Labeling Parameters and Their Associations with Risk Factors, Cerebral Small-Vessel Disease, and Etiologic Subtypes of Cognitive Impairment and Dementia. *AJNR Am J Neuroradiol, 43*(10), 1418-1423. https://doi.org/10.3174/ajnr.A7630

Gyanwali, B., Vrooman, H., Venketasubramanian, N., Wong, T. Y., Cheng, C. Y., Chen, C., & Hilal, S. (2019). Cerebral Small Vessel Disease and Enlarged Perivascular Spaces-Data From Memory Clinic and Population-Based Settings. *Front Neurol, 10*, 669. https://doi.org/10.3389/fneur.2019.00669

Han, H., Lin, Z., Soldan, A., Pettigrew, C., Betz, J. F., Oishi, K., Li, Y., Liu, P., Albert, M., & Lu, H. (2022, Nov). Longitudinal Changes in Global Cerebral Blood Flow in Cognitively Normal Older Adults: A Phase-Contrast MRI Study. *J Magn Reson Imaging, 56*(5), 1538-1545. https://doi.org/10.1002/jmri.28133

Han, H., Ning, Z., Yang, D., Yu, M., Qiao, H., Chen, S., Chen, Z., Li, D., Zhang, R., Liu, G., & Zhao, X. (2022, Aug). Associations between cerebral blood flow and progression of white matter hyperintensity in community-dwelling adults: a longitudinal cohort study. *Quant Imaging Med Surg, 12*(8), 4151-4165. https://doi.org/10.21037/qims-22-141

Hillmer, L., Erhardt, E. B., Caprihan, A., Adair, J. C., Knoefel, J. E., Prestopnik, J., Thompson, J., Hobson, S., & Rosenberg, G. A. (2022, Dec 15). Blood-brain barrier disruption measured by albumin index correlates with inflammatory fluid biomarkers. *J Cereb Blood Flow Metab*, 271678X221146127. https://doi.org/10.1177/0271678X221146127

Huang, C. J., Zhou, X., Yuan, X., Zhang, W., Li, M. X., You, M. Z., Zhu, X. Q., & Sun, Z. W. (2021). Contribution of Inflammation and Hypoperfusion to White Matter Hyperintensities-Related Cognitive Impairment. *Front Neurol, 12*, 786840. https://doi.org/10.3389/fneur.2021.786840

Huang, H., Zhao, K., Zhu, W., Li, H., & Zhu, W. (2021). Abnormal Cerebral Blood Flow and Functional Connectivity Strength in Subjects With White Matter Hyperintensities. *Front Neurol, 12*, 752762. https://doi.org/10.3389/fneur.2021.752762

Huisa, B. N., Caprihan, A., Thompson, J., Prestopnik, J., Qualls, C. R., & Rosenberg, G. A. (2015, Sep). Long-Term Blood-Brain Barrier Permeability Changes in Binswanger Disease. *Stroke, 46*(9), 2413-2418. https://doi.org/10.1161/STROKEAHA.115.009589

Huo, Y., Wang, Y., Guo, C., Liu, Q., Shan, L., Liu, M., Wu, H., Li, G., Lv, H., Lu, L., Zhou, Y., Feng, J., & Han, Y. (2023, Apr). Deep white matter hyperintensity is spatially correlated to MRI-visible perivascular spaces in cerebral small vessel disease on 7 Tesla MRI. *Stroke Vasc Neurol, 8*(2), 144-150. https://doi.org/10.1136/svn-2022-001611

Hurford, R., Charidimou, A., Fox, Z., Cipolotti, L., Jager, R., & Werring, D. J. (2014, May). MRI-visible perivascular spaces: relationship to cognition and small vessel disease MRI markers in ischaemic stroke and TIA. *J Neurol Neurosurg Psychiatry, 85*(5), 522-525. https://doi.org/10.1136/jnnp-2013-305815

Jann, K., Shao, X., Ma, S. J., Cen, S. Y., D'Orazio, L., Barisano, G., Yan, L., Casey, M., Lamas, J., Staffaroni, A. M., Kramer, J. H., Ringman, J. M., & Wang, D. J. J. (2021). Evaluation of Cerebral Blood Flow Measured by 3D PCASL as Biomarker of Vascular Cognitive Impairment and Dementia (VCID) in a Cohort of Elderly Latinx Subjects at Risk of Small Vessel Disease. *Front Neurosci, 15*, 627627. https://doi.org/10.3389/fnins.2021.627627

Kang, P., Ying, C., Chen, Y., Ford, A. L., An, H., & Lee, J. M. (2022, May). Oxygen Metabolic Stress and White Matter Injury in Patients With Cerebral Small Vessel Disease. *Stroke, 53*(5), 1570-1579. https://doi.org/10.1161/STROKEAHA.121.035674

Kerkhofs, D., Wong, S. M., Zhang, E., Staals, J., Jansen, J. F. A., van Oostenbrugge, R. J., & Backes, W. H. (2021, Apr 27). Baseline Blood-Brain Barrier Leakage and Longitudinal Microstructural Tissue Damage in the Periphery of White Matter Hyperintensities. *Neurology, 96*(17), e2192-e2200. https://doi.org/10.1212/WNL.0000000000011783

Kim, C. M., Alvarado, R. L., Stephens, K., Wey, H. Y., Wang, D. J. J., Leritz, E. C., & Salat, D. H. (2020, Feb). Associations between cerebral blood flow and structural and functional brain imaging measures in individuals with neuropsychologically defined mild cognitive impairment. *Neurobiol Aging, 86*, 64-74. https://doi.org/10.1016/j.neurobiolaging.2019.10.023

Kim, W. S. H., Luciw, N. J., Atwi, S., Shirzadi, Z., Dolui, S., Detre, J. A., Nasrallah, I. M., Swardfager, W., Bryan, R. N., Launer, L. J., & MacIntosh, B. J. (2022, Aug 15). Associations of white matter hyperintensities with networks of gray matter blood flow and volume in midlife adults: A coronary artery risk development in young adults magnetic resonance imaging substudy. *Hum Brain Mapp, 43*(12), 3680-3693. https://doi.org/10.1002/hbm.25876

Knoops, A. J., van der Graaf, Y., Appelman, A. P., Mali, W. P., & Geerlings, M. I. (2009, Oct). Total cerebral blood flow and hippocampal volume in patients with arterial disease. The SMART-MR study. *J Cereb Blood Flow Metab, 29*(10), 1727-1733. https://doi.org/10.1038/jcbfm.2009.91

Lau, K. K., Li, L., Lovelock, C. E., Zamboni, G., Chan, T. T., Chiang, M. F., Lo, K. T., Kuker, W., Mak, H. K., & Rothwell, P. M. (2017, Jun). Clinical Correlates, Ethnic Differences, and Prognostic Implications of Perivascular Spaces in Transient Ischemic Attack and Ischemic Stroke. *Stroke, 48*(6), 1470-1477. https://doi.org/10.1161/STROKEAHA.117.016694

Laveskog, A., Wang, R., Bronge, L., Wahlund, L. O., & Qiu, C. (2018, Jan). Perivascular Spaces in Old Age: Assessment, Distribution, and Correlation with White Matter Hyperintensities. *AJNR Am J Neuroradiol, 39*(1), 70-76. https://doi.org/10.3174/ajnr.A5455

Li, Y., Kalpouzos, G., Laukka, E. J., Dekhtyar, S., Backman, L., Fratiglioni, L., & Qiu, C. (2022, Apr). Progression of neuroimaging markers of cerebral small vessel disease in older adults: A 6-year follow-up study. *Neurobiol Aging, 112*, 204-211. https://doi.org/10.1016/j.neurobiolaging.2022.01.006

Li, Y., Li, M., Zhang, X., Shi, Q., Yang, S., Fan, H., Qin, W., Yang, L., Yuan, J., Jiang, T., & Hu, W. (2017, Jul). Higher blood-brain barrier permeability is associated with higher white matter hyperintensities burden. *J Neurol, 264*(7), 1474-1481. https://doi.org/10.1007/s00415-017-8550-8

Libecap, T. J., Zachariou, V., Bauer, C. E., Wilcock, D. M., Jicha, G. A., Raslau, F. D., & Gold, B. T. (2022). Enlarged Perivascular Spaces Are Negatively Associated With Montreal Cognitive Assessment Scores in Older Adults. *Front Neurol, 13*, 888511. https://doi.org/10.3389/fneur.2022.888511

Liem, M. K., Lesnik Oberstein, S. A., Haan, J., Boom, R., Ferrari, M. D., Buchem, M. A., & Grond, J. (2009, Jun). Cerebrovascular reactivity is a main determinant of white matter hyperintensity progression in CADASIL. *AJNR Am J Neuroradiol, 30*(6), 1244-1247. https://doi.org/10.3174/ajnr.A1533

Lin, C. Y., Jhan, S. R., Lee, W. J., Chen, P. L., Chen, J. P., Chen, H. C., & Chen, T. B. (2021). Imaging Markers of Subcortical Vascular Dementia in Patients With Multiple-Lobar Cerebral Microbleeds. *Front Neurol, 12*, 747536. https://doi.org/10.3389/fneur.2021.747536

Loos, C. M., Klarenbeek, P., van Oostenbrugge, R. J., & Staals, J. (2015). Association between Perivascular Spaces and Progression of White Matter Hyperintensities in Lacunar Stroke Patients. *PLoS One, 10*(9), e0137323. https://doi.org/10.1371/journal.pone.0137323

Lu, W., Yu, C., Wang, L., Wang, F., & Qiu, J. (2022). Perfusion heterogeneity of cerebral small vessel disease revealed via arterial spin labeling MRI and machine learning. *Neuroimage Clin, 36*, 103165. https://doi.org/10.1016/j.nicl.2022.103165

Main Abstracts. (2021, Sep). *Eur Stroke J, 6*(1 Suppl), 3-513. https://doi.org/10.1177/23969873211034932

Marstrand, J. R., Garde, E., Rostrup, E., Ring, P., Rosenbaum, S., Mortensen, E. L., & Larsson, H. B. (2002, Apr). Cerebral perfusion and cerebrovascular reactivity are reduced in white matter hyperintensities. *Stroke, 33*(4), 972-976. https://doi.org/10.1161/01.str.0000012808.81667.4b

Martinez-Ramirez, S., Pontes-Neto, O. M., Dumas, A. P., Auriel, E., Halpin, A., Quimby, M., Gurol, M. E., Greenberg, S. M., & Viswanathan, A. (2013, Apr 23). Topography of dilated perivascular spaces in subjects from a memory clinic cohort. *Neurology, 80*(17), 1551-1556. https://doi.org/10.1212/WNL.0b013e31828f1876

Munoz Maniega, S., Chappell, F. M., Valdes Hernandez, M. C., Armitage, P. A., Makin, S. D., Heye, A. K., Thrippleton, M. J., Sakka, E., Shuler, K., Dennis, M. S., & Wardlaw, J. M. (2017, Feb). Integrity of normal-appearing white matter: Influence of age, visible lesion burden and hypertension in patients with small-vessel disease. *J Cereb Blood Flow Metab, 37*(2), 644-656. https://doi.org/10.1177/0271678X16635657

Nasel, C., Boubela, R., Kalcher, K., & Moser, E. (2017, Feb). Normalised time-to-peak-distribution curves correlate with cerebral white matter hyperintensities - Could this improve early diagnosis? *J Cereb Blood Flow Metab, 37*(2), 444-455. https://doi.org/10.1177/0271678X16629485

Ni, L., Zhou, F., Qing, Z., Zhang, X., Li, M., Zhu, B., Zhang, B., & Xu, Y. (2020). The Asymmetry of White Matter Hyperintensity Burden Between Hemispheres Is Associated With Intracranial Atherosclerotic Plaque Enhancement Grade. *Front Aging Neurosci, 12*, 163. https://doi.org/10.3389/fnagi.2020.00163

Nylander, R., Fahlstrom, M., Rostrup, E., Kullberg, J., Damangir, S., Ahlstrom, H., Lind, L., & Larsson, E. M. (2018, May). Quantitative and qualitative MRI evaluation of cerebral small vessel disease in an elderly population: a longitudinal study. *Acta Radiol, 59*(5), 612-618. https://doi.org/10.1177/0284185117727567

O'Sullivan, M., Lythgoe, D. J., Pereira, A. C., Summers, P. E., Jarosz, J. M., Williams, S. C., & Markus, H. S. (2002, Aug 13). Patterns of cerebral blood flow reduction in patients with ischemic leukoaraiosis. *Neurology, 59*(3), 321-326. https://doi.org/10.1212/wnl.59.3.321

Pahlavian, S. H., Wang, X., Ma, S., Zheng, H., Casey, M., D'Orazio, L. M., Shao, X., Ringman, J. M., Chui, H., Wang, D. J., & Yan, L. (2021, Mar). Cerebroarterial pulsatility and resistivity indices are associated with cognitive impairment and white matter hyperintensity in elderly subjects: A phase-contrast MRI study. *J Cereb Blood Flow Metab, 41*(3), 670-683. https://doi.org/10.1177/0271678X20927101

Paradise, M. B., Beaudoin, M. S., Dawes, L., Crawford, J. D., Wen, W., Brodaty, H., & Sachdev, P. S. (2020, Feb 15). Development and validation of a rating scale for perivascular spaces on 3T MRI. *J Neurol Sci, 409*, 116621. https://doi.org/10.1016/j.jns.2019.116621

Phyu, P., Merwick, A., Davagnanam, I., Bolsover, F., Jichi, F., Wheeler-Kingshott, C., Golay, X., Hughes, D., Cipolotti, L., Murphy, E., Lachmann, R. H., & Werring, D. J. (2018, Apr 17). Increased resting cerebral blood flow in adult Fabry disease: MRI arterial spin labeling study. *Neurology, 90*(16), e1379-e1385. https://doi.org/10.1212/WNL.0000000000005330

Promjunyakul, N., Lahna, D., Kaye, J. A., Dodge, H. H., Erten-Lyons, D., Rooney, W. D., & Silbert, L. C. (2015). Characterizing the white matter hyperintensity penumbra with cerebral blood flow measures. *Neuroimage Clin, 8*, 224-229. https://doi.org/10.1016/j.nicl.2015.04.012

Promjunyakul, N. O., Dodge, H. H., Lahna, D., Boespflug, E. L., Kaye, J. A., Rooney, W. D., & Silbert, L. C. (2018, Jun 12). Baseline NAWM structural integrity and CBF predict periventricular WMH expansion over time. *Neurology, 90*(24), e2119-e2126. https://doi.org/10.1212/WNL.0000000000005684

Ramirez, J., Berezuk, C., McNeely, A. A., Scott, C. J., Gao, F., & Black, S. E. (2015). Visible Virchow-Robin spaces on magnetic resonance imaging of Alzheimer's disease patients and normal elderly from the Sunnybrook Dementia Study. *J Alzheimers Dis, 43*(2), 415-424. https://doi.org/10.3233/JAD-132528

Rane, S., Koh, N., Boord, P., Madhyastha, T., Askren, M. K., Jayadev, S., Cholerton, B., Larson, E., & Grabowski, T. J. (2018, May). Quantitative cerebrovascular pathology in a community-based cohort of older adults. *Neurobiol Aging, 65*, 77-85. https://doi.org/10.1016/j.neurobiolaging.2018.01.006

Rane, S., Owen, J., Hippe, D. S., Cholerton, B., Zabetian, C. P., Montine, T., & Grabowski, T. J. (2020, Nov). White Matter Lesions in Mild Cognitive Impairment and Idiopathic Parkinson's Disease: Multimodal Advanced MRI and Cognitive Associations. *J Neuroimaging, 30*(6), 843-850. https://doi.org/10.1111/jon.12778

Rodriguez Lara, F., Toro, A. R., Pinheiro, A., Demissie, S., Ekenze, O., Martinez, O., Parva, P., Charidimou, A., Ghosh, S., DeCarli, C., Seshadri, S., Habes, M., Maillard, P., & Romero, J. R. (2023, Sep 14). Relation of MRI-Visible Perivascular Spaces and Other MRI Markers of Cerebral Small Vessel Disease. *Brain Sci, 13*(9). https://doi.org/10.3390/brainsci13091323

Sachdev, P., Wen, W., Shnier, R., & Brodaty, H. (2004, Winter). Cerebral blood volume in T2-weighted white matter hyperintensities using exogenous contrast based perfusion MRI. *J Neuropsychiatry Clin Neurosci, 16*(1), 83-92. https://doi.org/10.1176/jnp.16.1.83

Sahin, N., Solak, A., Genc, B., & Akpinar, M. B. (2015, Jul). Dilatation of the Virchow-Robin spaces as an indicator of unilateral carotid artery stenosis: correlation with white matter lesions. *Acta Radiol, 56*(7), 852-859. https://doi.org/10.1177/0284185114544243

Salomonsson, T., Rumetshofer, T., Jonsen, A., Bengtsson, A. A., Zervides, K. A., Nilsson, P., Knutsson, M., Wirestam, R., Latt, J., Knutsson, L., & Sundgren, P. C. (2023). Abnormal cerebral hemodynamics and blood-brain barrier permeability detected with perfusion MRI in systemic lupus erythematosus patients. *Neuroimage Clin, 38*, 103390. https://doi.org/10.1016/j.nicl.2023.103390

Sam, K., Crawley, A. P., Poublanc, J., Conklin, J., Sobczyk, O., Mandell, D. M., Duffin, J., Venkatraghavan, L., Fisher, J. A., Black, S. E., & Mikulis, D. J. (2016, Dec). Vascular Dysfunction in Leukoaraiosis. *AJNR Am J Neuroradiol, 37*(12), 2258-2264. https://doi.org/10.3174/ajnr.A4888

Shams, S., Martola, J., Charidimou, A., Larvie, M., Granberg, T., Shams, M., Kristoffersen-Wiberg, M., & Wahlund, L. O. (2017, Sep 22). Topography and Determinants of Magnetic Resonance Imaging (MRI)-Visible Perivascular Spaces in a Large Memory Clinic Cohort. *J Am Heart Assoc, 6*(9). https://doi.org/10.1161/JAHA.117.006279

Shao, X., Jann, K., Ma, S. J., Yan, L., Montagne, A., Ringman, J. M., Zlokovic, B. V., & Wang, D. J. J. (2020). Comparison Between Blood-Brain Barrier Water Exchange Rate and Permeability to Gadolinium-Based Contrast Agent in an Elderly Cohort. *Front Neurosci, 14*, 571480. https://doi.org/10.3389/fnins.2020.571480

Shao, X., Ma, S. J., Casey, M., D'Orazio, L., Ringman, J. M., & Wang, D. J. J. (2019, May). Mapping water exchange across the blood-brain barrier using 3D diffusion-prepared arterial spin labeled perfusion MRI. *Magn Reson Med, 81*(5), 3065-3079. https://doi.org/10.1002/mrm.27632

Shen, M., Wei, G., Cheng, M., & Jiang, H. (2020, Apr). Association between Enlarged Perivascular Spaces and Internal Carotid Artery Stenosis: A Study in Patients Diagnosed by Digital Subtraction Angiography. *J Stroke Cerebrovasc Dis, 29*(4), 104635. https://doi.org/10.1016/j.jstrokecerebrovasdis.2019.104635

Shi, L., Miao, X., Lou, W., Liu, K., Abrigo, J., Wong, A., Chu, W. C. W., Wang, D., & Mok, V. C. T. (2017). The Spatial Associations of Cerebral Blood Flow and Spontaneous Brain Activities with White Matter Hyperintensities-An Exploratory Study Using Multimodal Magnetic Resonance Imaging. *Front Neurol, 8*, 593. https://doi.org/10.3389/fneur.2017.00593

Song, S. K., Lee, S. K., Lee, J. J., Lee, J. E., Choi, H. S., Sohn, Y. H., & Lee, P. H. (2011, Dec). Blood-brain barrier impairment is functionally correlated with clinical severity in patients of multiple system atrophy. *Neurobiol Aging, 32*(12), 2183-2189. https://doi.org/10.1016/j.neurobiolaging.2009.12.017

Staffaroni, A. M., Cobigo, Y., Elahi, F. M., Casaletto, K. B., Walters, S. M., Wolf, A., Lindbergh, C. A., Rosen, H. J., & Kramer, J. H. (2019, Aug 15). A longitudinal characterization of perfusion in the aging brain and associations with cognition and neural structure. *Hum Brain Mapp, 40*(12), 3522-3533. https://doi.org/10.1002/hbm.24613

Starr, J. M., Wardlaw, J., Ferguson, K., MacLullich, A., Deary, I. J., & Marshall, I. (2003, Jan). Increased blood-brain barrier permeability in type II diabetes demonstrated by gadolinium magnetic resonance imaging. *J Neurol Neurosurg Psychiatry, 74*(1), 70-76. https://doi.org/10.1136/jnnp.74.1.70

Tarumi, T., Ayaz Khan, M., Liu, J., Tseng, B. Y., Parker, R., Riley, J., Tinajero, C., & Zhang, R. (2014, Jun). Cerebral hemodynamics in normal aging: central artery stiffness, wave reflection, and pressure pulsatility. *J Cereb Blood Flow Metab, 34*(6), 971-978. https://doi.org/10.1038/jcbfm.2014.44

Thanprasertsuk, S., Martinez-Ramirez, S., Pontes-Neto, O. M., Ni, J., Ayres, A., Reed, A., Swords, K., Gurol, M. E., Greenberg, S. M., & Viswanathan, A. (2014, Aug 26). Posterior white matter disease distribution as a predictor of amyloid angiopathy. *Neurology, 83*(9), 794-800. https://doi.org/10.1212/WNL.0000000000000732

Tu, M. C., Chung, H. W., Hsu, Y. H., Yang, J. J., & Wu, W. C. (2022). Stage-Dependent Cerebral Blood Flow and Leukoaraiosis Couplings in Subcortical Ischemic Vascular Disease and Alzheimer's Disease. *J Alzheimers Dis, 86*(2), 729-739. https://doi.org/10.3233/JAD-215405

Uh, J., Yezhuvath, U., Cheng, Y., & Lu, H. (2010, Jul). In vivo vascular hallmarks of diffuse leukoaraiosis. *J Magn Reson Imaging, 32*(1), 184-190. https://doi.org/10.1002/jmri.22209

van Dalen, J. W., Mutsaerts, H., Nederveen, A. J., Vrenken, H., Steenwijk, M. D., Caan, M. W. A., Majoie, C., van Gool, W. A., & Richard, E. (2016, Oct). White Matter Hyperintensity Volume and Cerebral Perfusion in Older Individuals with Hypertension Using Arterial Spin-Labeling. *AJNR Am J Neuroradiol, 37*(10), 1824-1830. https://doi.org/10.3174/ajnr.A4828

van der Veen, P. H., Muller, M., Vincken, K. L., Hendrikse, J., Mali, W. P., van der Graaf, Y., Geerlings, M. I., & Group, S. S. (2015, May). Longitudinal relationship between cerebral small-vessel disease and cerebral blood flow: the second manifestations of arterial disease-magnetic resonance study. *Stroke, 46*(5), 1233-1238. https://doi.org/10.1161/STROKEAHA.114.008030

van Es, A. C., van der Grond, J., ten Dam, V. H., de Craen, A. J., Blauw, G. J., Westendorp, R. G., Admiraal-Behloul, F., van Buchem, M. A., & Group, P. S. (2010, Mar 23). Associations between total cerebral blood flow and age related changes of the brain. *PLoS One, 5*(3), e9825. https://doi.org/10.1371/journal.pone.0009825

Wang, M. L., Yu, M. M., Wei, X. E., Li, W. B., Li, Y. H., & Alzheimer's Disease Neuroimaging, I. (2021, Apr). Association of enlarged perivascular spaces with Abeta and tau deposition in cognitively normal older population. *Neurobiol Aging, 100*, 32-38. https://doi.org/10.1016/j.neurobiolaging.2020.12.014

Wang, R., Zhang, J., Shang, J., Wang, F., & Yan, X. (2022, Aug 28). Effects of different regional cerebral blood flow on white matter hyperintensity in CADASIL patients. *J Biomed Res, 36*(5), 368-374. https://doi.org/10.7555/JBR.36.20220006

Wang, S., Huang, P., Zhang, R., Hong, H., Jiaerken, Y., Lian, C., Yu, X., Luo, X., Li, K., Zeng, Q., Xu, X., Yu, W., Wu, X., & Zhang, M. (2021, Oct). Quantity and Morphology of Perivascular Spaces: Associations With Vascular Risk Factors and Cerebral Small Vessel Disease. *J Magn Reson Imaging, 54*(4), 1326-1336. https://doi.org/10.1002/jmri.27702

Wang, X., Valdes Hernandez Mdel, C., Doubal, F., Chappell, F. M., Piper, R. J., Deary, I. J., & Wardlaw, J. M. (2016, Jan 15). Development and initial evaluation of a semi-automatic approach to assess perivascular spaces on conventional magnetic resonance images. *J Neurosci Methods, 257*, 34-44. https://doi.org/10.1016/j.jneumeth.2015.09.010

Wang, X. Y., Lyu, J. H., Zhang, S. H., Duan, C. H., Duan, Q., Ma, X. X., Zhang, T. Y., Zhang, J., Tian, C. L., & Lou, X. (2022, Jul). Severity of Intracranial Large Artery Disease Correlates With Cerebral Small Vessel Disease. *J Magn Reson Imaging, 56*(1), 264-272. https://doi.org/10.1002/jmri.28004

Xia, Y., Shen, Y., Wang, Y., Yang, L., Wang, Y., Li, Y., Liang, X., Zhao, Q., Wu, J., Chu, S., Liang, Z., Wang, X., Qiu, B., Ding, H., Ding, D., Cheng, X., & Dong, Q. (2020, May 10). White matter hyperintensities associated with progression of cerebral small vessel disease: a 7-year Chinese urban community study. *Aging (Albany NY), 12*(9), 8506-8522. https://doi.org/10.18632/aging.103154

Yakushiji, Y., Charidimou, A., Hara, M., Noguchi, T., Nishihara, M., Eriguchi, M., Nanri, Y., Nishiyama, M., Werring, D. J., & Hara, H. (2014, Dec 2). Topography and associations of perivascular spaces in healthy adults: the Kashima scan study. *Neurology, 83*(23), 2116-2123. https://doi.org/10.1212/WNL.0000000000001054

Yamada, S., Ishikawa, M., Yamamoto, K., Yamaguchi, M., & Oshima, M. (2019, Mar 15). Location-specific characteristics of perivascular spaces as the brain's interstitial fluid drainage system. *J Neurol Sci, 398*, 9-15. https://doi.org/10.1016/j.jns.2019.01.022

Yao, M., Herve, D., Jouvent, E., Duering, M., Reyes, S., Godin, O., Guichard, J. P., Dichgans, M., & Chabriat, H. (2014). Dilated perivascular spaces in small-vessel disease: a study in CADASIL. *Cerebrovasc Dis, 37*(3), 155-163. https://doi.org/10.1159/000356982

Yao, M., Zhu, Y. C., Soumare, A., Dufouil, C., Mazoyer, B., Tzourio, C., & Chabriat, H. (2014, Sep). Hippocampal perivascular spaces are related to aging and blood pressure but not to cognition. *Neurobiol Aging, 35*(9), 2118-2125. https://doi.org/10.1016/j.neurobiolaging.2014.03.021

Zeng, Q., Li, K., Luo, X., Wang, S., Xu, X., Jiaerken, Y., Liu, X., Hong, L., Hong, H., Li, Z., Fu, Y., Zhang, T., Chen, Y., Liu, Z., Huang, P., Zhang, M., & for behalf of Alzheimer's Disease Neuroimaging, I. (2022, Aug). The association of enlarged perivascular space with microglia-related inflammation and Alzheimer's pathology in cognitively normal elderly. *Neurobiol Dis, 170*, 105755. https://doi.org/10.1016/j.nbd.2022.105755

Zhang, C. E., Wong, S. M., Uiterwijk, R., Backes, W. H., Jansen, J. F. A., Jeukens, C., van Oostenbrugge, R. J., & Staals, J. (2019, Apr). Blood-brain barrier leakage in relation to white matter hyperintensity volume and cognition in small vessel disease and normal aging. *Brain Imaging Behav, 13*(2), 389-395. https://doi.org/10.1007/s11682-018-9855-7

Zhang, K., Zhou, Y., Zhang, W., Li, Q., Sun, J., & Lou, M. (2022, Jan). MRI-visible perivascular spaces in basal ganglia but not centrum semiovale or hippocampus were related to deep medullary veins changes. *J Cereb Blood Flow Metab, 42*(1), 136-144. https://doi.org/10.1177/0271678X211038138

Zhang, Q., Datta, R., Detre, J. A., & Cucchiara, B. (2017, May). White matter lesion burden in migraine with aura may be associated with reduced cerebral blood flow. *Cephalalgia, 37*(6), 517-524. https://doi.org/10.1177/0333102416649760

Zhang, R., Huang, P., Wang, S., Jiaerken, Y., Hong, H., Zhang, Y., Yu, X., Lou, M., & Zhang, M. (2022). Decreased Cerebral Blood Flow and Delayed Arterial Transit Are Independently Associated With White Matter Hyperintensity. *Front Aging Neurosci, 14*, 762745. https://doi.org/10.3389/fnagi.2022.762745

Zhang, W., Li, M., Zhou, X., Huang, C., Wan, K., Li, C., Yin, J., Zhao, W., Zhang, C., Zhu, X., & Sun, Z. (2022). Altered serum amyloid beta and cerebral perfusion and their associations with cognitive function in patients with subcortical ischemic vascular disease. *Frontiers in Neuroscience, 16*. https://doi.org/10.3389/fnins.2022.993767

Zhu, Y. C., Tzourio, C., Soumare, A., Mazoyer, B., Dufouil, C., & Chabriat, H. (2010, Nov). Severity of dilated Virchow-Robin spaces is associated with age, blood pressure, and MRI markers of small vessel disease: a population-based study. *Stroke, 41*(11), 2483-2490. https://doi.org/10.1161/STROKEAHA.110.591586
